# Association between sepsis incidence and regional socioeconomic deprivation and health care capacity in Germany – an ecological study

**DOI:** 10.1186/s12889-021-11629-4

**Published:** 2021-09-07

**Authors:** Norman Rose, Claudia Matthäus-Krämer, Daniel Schwarzkopf, André Scherag, Sebastian Born, Konrad Reinhart, Carolin Fleischmann-Struzek

**Affiliations:** 1 Center for Sepsis Control and Care, Jena University Hospital, Bachstraße 18, 07743 Jena, Germany; 2 Institute of Infectious Diseases and Infection Control, Jena University Hospital, Am Klinikum 1, 07747 Jena, Germany; 3 Department for Anesthesiology and Intensive Care Medicine, Jena University Hospital, Am Klinikum 1, 07740 Jena, Germany; 4 Institute of Medical Statistics, Computer and Data Sciences, Jena University Hospital, Bachstraße 18, 07743 Jena, Germany; 5 Department of Anesthesiology and Intensive Care Medicine, Charité Universitätsmedizin Berlin, Charitéplatz 1, 10117 Berlin, Germany

**Keywords:** Sepsis, Incidence, Ecological study, Socioeconomic factors, Medical services

## Abstract

**Background:**

Sepsis is a substantial health care burden. Data on regional variation in sepsis incidence in Germany and any possible associations with regional socioeconomic deprivation and health care capacity is lacking.

**Methods:**

Ecological study based on the nationwide hospital Diagnosis-related Groups (DRG) statistics data of 2016. We identified sepsis by ICD-10-codes and calculated crude and age-standardized incidence proportions in the 401 administrative German districts. Associations between socioeconomic and health care capacity indicators and crude and age-adjusted sepsis incidence were investigated by simple and multiple negative binomial (NB) regressions.

**Results:**

In 2016, sepsis incidence was 178 per 100,000 inhabitants and varied 10-fold between districts. We found that the rate of students leaving school without certificate was significantly associated with crude and age-standardized explicit sepsis incidence in the simple and multiple NB regressions. While we observed no evidence for an association to the capacity of hospital beds and general practitioners, the distance to the nearest pharmacy was associated with crude- and age-standardized sepsis incidence. In the multiple regression analyses, an increase of the mean distance + 1000 m was associated with an expected increase by 21.6 [95% CI, 10.1, 33.0] (*p* < 0.001), and 11.1 [95% CI, 1.0, 21.2]/100,000 population (*p* = .026) after adjusting for age differences between districts.

**Conclusions:**

Residence in districts with lower socioeconomic status (e.g., less education) and further distance to pharmacies are both associated with an increased sepsis incidence. This warrants further research with individual-level patient data to better model and understand such dependencies and to ultimately design public health interventions to address the burden of sepsis in Germany.

**Supplementary Information:**

The online version contains supplementary material available at 10.1186/s12889-021-11629-4.

## Introduction

Sepsis is the body’s dysregulated response to infection resulting in life-threatening organ dysfunction [1]. It affects an estimated 49.8 million patients worldwide annually and is associated with 19.2% of deaths [2]. Sepsis is an emergency that requires timely diagnosis and urgent medical treatment [3]. Elderly patients, those with chronic health conditions [4], asplenia [5], or immunosuppressive therapies [6] are at increased risk for sepsis and accompanying adverse short- and long-term outcomes [7].

Previous work has demonstrated that in the United States (US), residence in medically underserved or socioeconomically deprived regions is associated with an increased sepsis incidence [8, 9]. It has been hypothesized that this might be due to the poorer access to prevention and health care services in these regions, which may lead to inadequate management of chronic conditions and a critical delay in initial evaluation and treatment of infections associated with higher risks to progression into sepsis [8, 10]. Other studies suggest that stress may act as the link between regional socioeconomic deprivation and health risk (e.g., stress due to factors such as overcrowding, poor infrastructure, a lack of social support) [11]. However, data supporting the generalizability of these findings to other countries are lacking.

In Germany, the incidence proportion (sepsis cases per inhabitants, denoted as incidence in the following) of sepsis was 158/100,000 inhabitants in 2015 [12]. The extent of regional differences in the sepsis incidence in Germany is unknown. Likewise, we lack information if regional differences in sepsis incidence can in part be attributed to regional socioeconomic deprivation or to structural variation of medical services in Germany. Although Germany has a widely accessible public health care and social security system [13], the association between regional deprivation and the occurrence of chronic and acute diseases has been demonstrated for various other conditions such as diabetes [14], cancer [15], and myocardial infarction [16], as well as for appendectomies [17].

The aim of this ecological study was to describe regional variation in hospital-treated sepsis incidence in Germany, and to investigate its association with regional socioeconomic deprivation and structural variation of medical services.

## Methods

This study was approved by the institutional review board of the Friedrich Schiller University Jena (#2018–1122-Daten).

### Study design and data source

We performed a retrospective ecological study based on the nationwide Diagnosis-related Groups (DRG) statistics of 2016. The DRG statistics is the largest all-payer inpatient database in Germany. Data collection is mandated by the Hospital Reimbursement Act §21 for all acute-care hospitals in Germany except for prison hospitals and psychiatric facilities. Each hospitalization is listed with primary and secondary International Classification of Diseases, Tenth Revision, German Modification (ICD-10-GM) codes, procedural codes, discharge disposition, patient demographics, and hospital length of stay. Furthermore, we used regional indicators provided in the INKAR (indicators and maps on spatial and urban development) database of the German Federal Institute for Research on Building, Urban Affairs and Spatial Development. Indicators were extracted on a district level and merged with the DRG statistics using the official municipality key of the patients’ residence included in both databases.

### Study sample and characteristics

Among all hospitalizations in 2016 in Germany, we identified hospitalizations with explicitly coded sepsis using the ICD-10-GM codes R65.1 (severe sepsis) and R57.2 (septic shock). In 2016, these codes were defining sepsis according to the sepsis-1 definition as sepsis with organ dysfunction (severe sepsis) [18]. Additionally, we used an alternative implicit approach, which is known as Angus implementation [19]. It identifies sepsis by the combination of ICD-10-GM codes for infection and organ failures (supplementary file 1) and thereby captures cases in which sepsis was not explicitly coded at hospital discharge. This approach is considered less prone to external coding incentives such as reimbursement for higher patient complexity. We characterized sepsis patients by comorbidity as defined by the Charlson Comorbidty Index [20], surgical treatment (any procedural code from chapter 5 = surgical procedures), intensive care treatment (procedural codes 8–980, 8-98d, 8-98f for intensive care complex treatment), hospital length of stay, hospital death and discharge to hospice (discharge disposition code = 7 or 11 in the DRG statistics, respectively).

### Regional classification and district-level predictors

Germany has 16 federal states and 401 districts, which form its administrative units. Sepsis incidence was calculated according to the patients’ place of residence on federal state and district level, which are coded by the official municipality key in the DRG statistics.

We chose three commonly used district level socioeconomic deprivation indicators [21–25] reflecting average occupation, income and education of the population for the analysis of contextual socioeconomic effects: The unemployment rate 2016 (proportion of unemployed among working age residents in %), the net household income 2016 (average household income in EUR per inhabitant), and the rate of school leavers without certificate 2016 (percent of students leaving school without having passed the lowest qualification certificate, the „Hauptschulabschluss “after 9 or 10 years of education, out of all students leaving school). Furthermore, we selected three indicators of inpatient and outpatient health care capacity and the density of medical services: hospital beds per 1000 inhabitants in 2016, general practitioner (GP) per 100,000 inhabitants in 2016, and straight line distance to the nearest pharmacy per inhabitant in meters as surrogate for the geographical proximity of medical services in 2017 (no 2016 data available). The definition of the airline distance to the nearest pharmacy is explained in the supplementary file 1.

Age has proved an important risk factor for sepsis [26]. To assess the relationship between age and sepsis incidence, we used the mean age of the districts’ population in 2016 provided in the INKAR database as predictor for the crude sepsis incidence.

### Statistical analyses

Regional differences in the crude and age-standardized incidence of sepsis between German districts were tested in a first step using a *χ*^2^-Test proposed by Snijders & Bosker [27]. The intraclass correlation (ICC) was computed as an effect size measure of the between-district heterogeneity. Negative binomial (NB) regression models were used in a second step to explain between-district variance in the incidence proportions by indicators of socioeconomic deprivation and health care capacity. The NB regression model was preferred over the Poisson regression model due to significant overdispersion in our data (supplementary file 2 Tables 1 and 2), which was tested using likelihood ratio tests. The NB models were fitted to (a) the number of sepsis hospitalizations per district, and (b) the age-standardized expected numbers of sepsis hospitalizations per district calculated by the direct method with Germany’s overall population in 2016 as reference. The number of inhabitants per district was taken into account as an offset variable in the model to account for differences in numbers of inhabitants between districts. First, each district level predictor was analyzed individually in a simple regression model predicting crude and age-standardized sepsis incidences. Some of the predictor variables are substantially correlated (Table 1). In order to estimate the unique contribution of the socioeconomic and medical service indicators, we used three multiple NB regressions for each outcome in a second step: (a) the multiple NB regression with the three indicators of socioeconomic deprivation; (b) the multiple NB regression with the three indicators of health care capacity; and (c) the multiple NB regression with all predictor variables. The last model served for statistical testing of the uniquely explained variance by socioeconomic deprivation and health care capacity indicators using the likelihood ratio test. Nagelkerke’s Pseudo-*R*^*2*^ was used as an effect size measure of the relationship between predictors and the incidence rates. The number of hospitals beds was unavailable for two German districts (Soemmerda and Fuerth), which were excluded from regression models that include this predictor variable.

**Table 1:** Pearson correlation coefficients of indicators of socioeconomic deprivation and medical infrastructure and mean age

	Unemployment rate	Net household income (100 Euro)	Rate of school leavers w/o certificate	Hospital beds/1000 population	GPs/100,000 population	Distance to the next pharmacy (1000 m)
Net household income (100 Euro/year)	−0.660***					
Rate of school leavers w/o certificate	0.485***	−0.461***				
Hospital beds/1000 population ^a^	0.315***	−0.195***	0.112*			
GPs/100,000 population	0.355***	−0.113*	0.111*	0.780***		
Distance to the next pharmacy (1000 m)	−0.256***	− 0.122*	0.108*	− 0.406***	− 0.627***	
Mean Age	0.334***	−0.350***	0.425***	−0.082	− 0.175***	0.408*

* *p* < 0.05, ** *p* < 0.01, *** *p* < 0.001

^a^ Correlation coefficients that include net household income are based on 399 instead of 401 German districts due to missing values for two districts (Soemmerda & Fuerth)

To illustrate the strength of the relationship between predictors and outcomes in common metrics, the parameters of the NB regressions were used to estimate the expected percentage change (*EPC*) as well as the expected change (*EC*) in the number of sepsis hospitalizations associated with an increase in the respective predictor variable (see supplementary file 3). In case of multiple NB regressions, EPC and EC are estimated under statistical control of the other predictors in the model and therefore can be interpreted as adjusted EPC and EC.

We report point estimates and interval estimates with 95% coverage in addition to two-sided *p*-values. The significance level was α = 0.05. All statistical analyses and computations were conducted via remote data processing using R [28], including the R functions ‘nagelkerke’ from the r package ‘rcompanion’ [29] and ‘glm.nb’ from the r package MASS [30]. Maps were created using the ‘spplot’ function from the ‘sp’ package [31, 32].

## Results

**Fig. 1:**
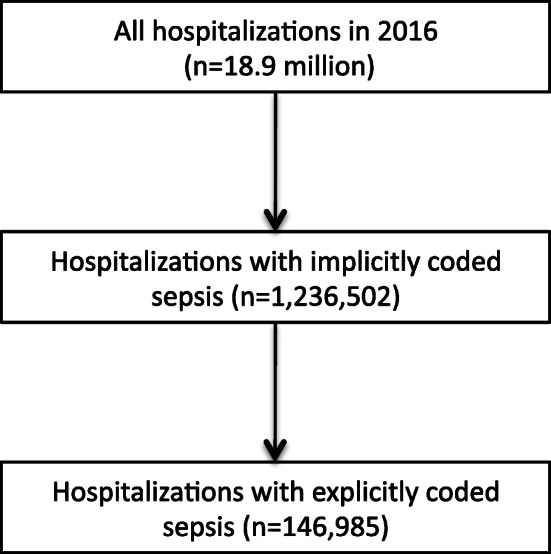
Flow of study inclusion

**Fig. 2:**
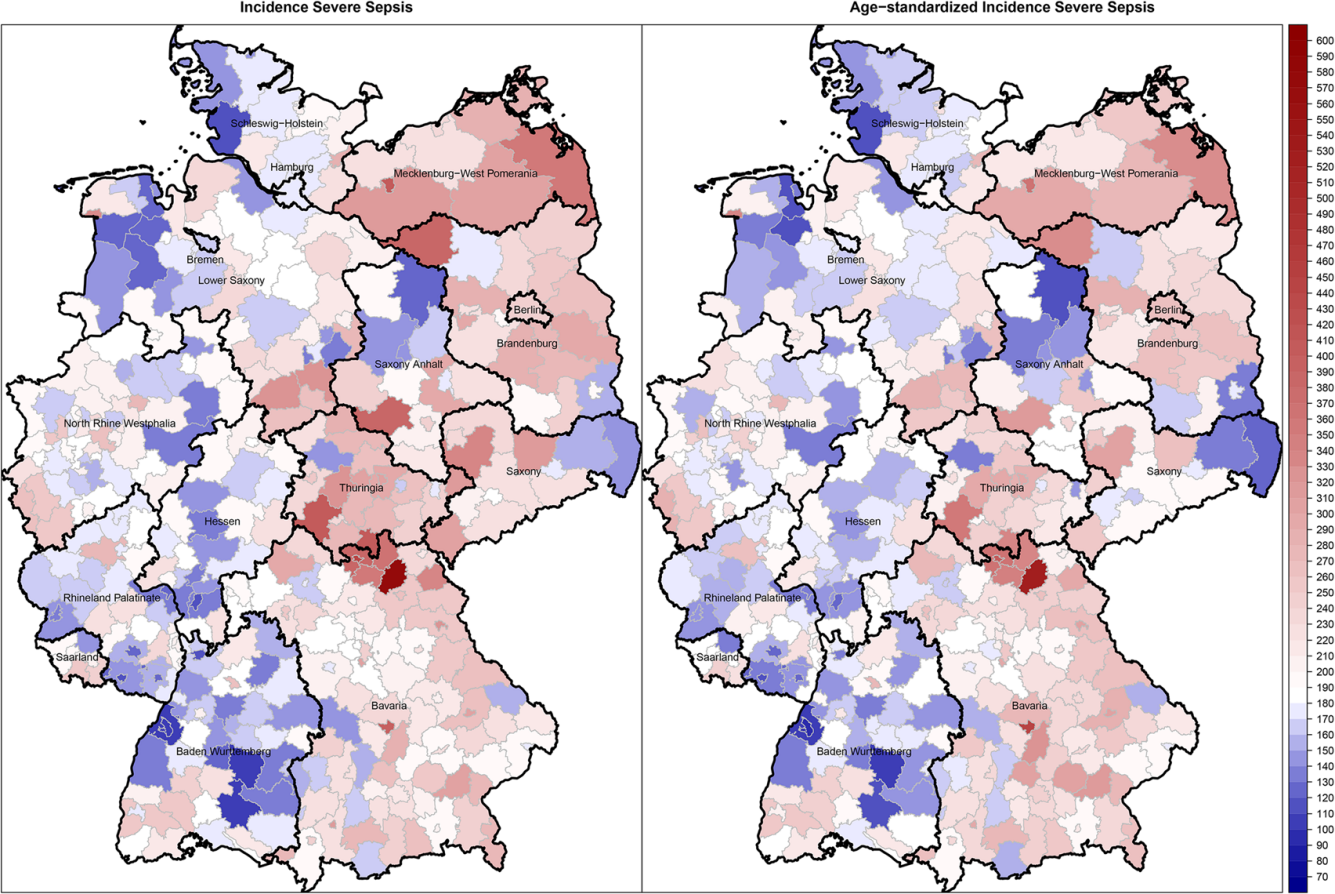
Distribution of crude and age-standardized explicitly defined sepsis (R65.1 – severe sepsis, R57.2 septic shock according to the 1992 sepsis-1 definitions [18]) incidence across German districts. Maps were created using the ‘spplot’ function from the ‘sp’ package [31, 32]. Geodata and shapefiles for creating maps of Germany in R were retrieved from https://gadm.org/. The maps are freely available for academic use

**Fig. 3:**
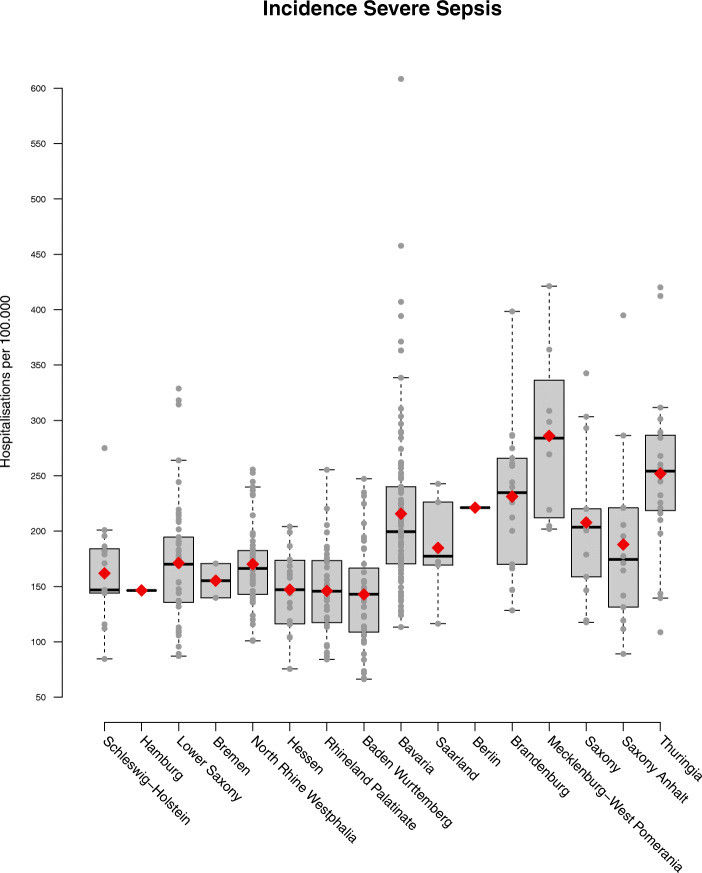
Distribution of explicitly defined sepsis incidence across German federal states and districts

Among 18.9 million hospitalizations, we identified 146,985 hospitalizations with sepsis explicitly coded at hospital discharge in 2016 (0.78% of all hospitalizations, Fig. 1). Demographics and clinical characteristics are provided in the supplementary file 2 Table 3. The overall sepsis incidence in 2016 was 178/100,000. On district level, the sepsis incidence ranged between 66 and 608 with a median of 174 and an interquartile range (IQR) of 143 to 218/100,000 population. The age-standardized sepsis incidence ranged from 57 to 550/100,000 population (median = 171, IQR = 142 to 212, Figs. 2, 3). We found a small but significant heterogeneity in the proportion of sepsis hospitalizations across German districts (ICC explicit sepsis = 0.02%, ICC age-standardized explicit sepsis = 0.02%, each *p* = 0.002).

The indicators of socioeconomic deprivation and medical infrastructure also showed differences between federal states and districts (Table 2, supplementary file 2 Table 4a and b, Figures 1–7). We found substantial Pearson correlation coefficients between *| r |* = 0.46 and | *r* | = 0.66 among the three socioeconomic indicators as well as among the three indicators for medical infrastructure (*| r |* = 0.41 to |* r* | = 0.78) (Table 1). However, the indicators of socioeconomic deprivation were only weakly correlated (absolute values: | *r* | = 0.11 to | *r* | = 0.36) with health capacity indicators. Mean age was weakly to moderately correlated (absolute values: | *r* | = 0.18 to | *r* | = 0.43) with all predictors except the number of hospital beds (*r* = − 0.08).

**Table 2:** Mean age, socioeconomic status and health care capacity among 401 German districts

Predictor	M	Median	SD	Min	Max
Mean Age	44.45	44.24	1.93	39.73	49.99
Unemployment rate	5.77	5.40	2.60	1.40	14.70
Net household income (100 Euro) ^a^	18.07	17.95	2.05	13.45	29.04
Rate of school leavers w/o certificate	5.97	5.60	2.09	1.20	14.19
Hospital beds/1000 population	6.39	5.47	3.88	0.00	29.59
GPs/100,000 population	60.71	52.42	25.60	8.34	153.15
Distance to the next pharmacy (1000 m)	1.50	1.49	0.78	0.35	3.82

^a^ Descriptive statistics of net household income are based on 399 German districts only, because of missing values for two districts (Soemmerda & Fuerth)

### Associations between regional characteristics and sepsis incidence

In the simple regression analyses, we found that sepsis incidence was significantly associated with the mean population age at the district level (Table 3). The expected change was *EC* = 12.6 [95% CI, 9.4, 15.8] (*p* < .001). Hence, two randomly selected districts that differ by 1 year in the mean age have an expected difference of 12.6 sepsis hospitalizations per 100,000 population. Furthermore, all socioeconomic indicators and the distance to the nearest pharmacy were statistically significant predictors of sepsis incidence in the simple regression analyses, whereas we observed no evidence for such an association to the number of hospital beds and the number of GPs. The mean age was found to be the strongest single predictor for the number of sepsis hospitalizations in terms of Pseudo-R^2^ (explicit: Pseudo-*R*^*2*^ = 0.140, implicit: Pseudo-*R*^*2*^ = 0.242).

**Table 3:** Results of the simple and multiple negative binomial regression analyses for the outcome crude and age-standardized incidence of sepsis (explicit) per 100.000 population

Predictor	Simple negative binomial regression			Multiple negative binomial regression			Multiple negative binomial regression (Full model)		
	*EC* (95% CI)	p	R^2^	*EC* (95% CI)	p	R^2^	*EC* (95% CI)	p	R^2^
**PANEL A: Outcome - crude sepsis incidence**									
Mean age	12.6 (9.4, 15.8)	< 0.001	0.140	–	–	–	–	–	–
*Socioeconomic indicators*									
Unemployment rate	2.5 (0.1, 4.9)	0.040	0.011	−2.1 (−5.3, 1.1)	0.196	0.057	−0.3 (−3.9, 3.4)	0.888	0.078
Net household income^a,c^	−5.1 (−8.0, − 2.2)	0.001	0.030	−4.0 (− 8.0, − 0.1)	0.046		−1.6 (− 5.8, 2.7)	0.477	
Rate of school leavers w/o certificate	6.5 (3.5, 9.6)	< 0.001	0.047	5.9 (2.4, 9.4)	0.001		4.9 (1.4, 8.5)	0.006	
*Indicators of medical infrastructure*									
Hospital beds/1000 population	1.2 (−0.4, 2.8)	0.142	0.057	2.0 (−0.5, 4.6)	0.120	0.096	1.8 (−0.8, 4.4)	0.164	
GPs/100,000 population	0.0 (−0.2, 0.3)	0.891	0.000	0.2 (−0.3, 0.6)	0.456		0.0 (−0.4, 0.5)	0.858	
Distance to the next pharmacy^b^	13.2 (4.6, 21.7)	0.002	0.023	21.6 (10.1, 33.0)	< 0.001		16.0 (3.9, 28.0)	0.007	
**PANEL B: Outcome - age-adjusted sepsis incidence**									
*Socioeconomic indicators*									
Unemployment rate	−0.1 (−2.3, 2.0)	0.901	0.000	−3.0 (−5.9, 0.0)	0.050	0.020	−2.9 (−6.3, 0.5)	0.095	0.030
Net household income^a,c^	−2.0 (−4.7, 0.8)	0.161	0.005	−2.8 (−6.5, 0.8)	0.133		−2.0 (− 6.0, 2.0)	0.330	
Rate of school leavers w/o certificate	2.8 (0.0, 5.5)	0.045	0.010	3.3 (0.1, 6.5)	0.041		2.9 (−0.4, 6.2)	0.078	
*Indicators of medical infrastructure*									
Hospital beds/1000 population	1.1 (−0.4, 2.6)	0.133	0.056	1.4 (− 1.0, 3.8)	0.245	0.068	1.4 (−1.0, 3.8)	0.248	
GPs/100,000 population	0.1 (−0.1, 0.3)	0.495	0.001	0.1 (−0.3, 0.5)	0.565		0.1 (−0.3, 0.5)	0.668	
Distance to the next pharmacy^b^	5.4 (−2.1, 12.9)	0.152	0.005	11.1 (1.0, 21.2)	0.026		6.4 (−4.4, 17.1)	0.238	

*Abbreviations*: *EC* Expected change, *w/o* without, *CI* Confidence Interval

^a^ Increase per 100 Euro

^b^ Increase per 1000 m

^c^ Simple and multiple negative binomial regression models include net household income as a predictor are based on 399 instead of 401 German districts due to missing values for two districts (Soemmerda & Fuerth)

Adjusting for all other predictors in the multiple regression analyses, effects of mean household income and rate of school leavers without certificate on sepsis incidence were attenuated, but still significant. Unemployment rate was not significantly associated with the sepsis incidence given the other two predictors. Positive associations were also found between age-adjusted sepsis incidence and the rate of school leavers without certificate, although they were weaker compared to the associations with unadjusted sepsis incidence (Table 3). The adjusted *EC* in the age-standardized sepsis incidence for the rate of school leavers without certificate was *EC* = 3.3 [95% CI, 0.1, 6.5] (*p* = .041). Hence, two randomly selected districts with equal age distributions that differ by 1% in the proportion of school leavers, but no differences in the other predictors in the model have an expected difference of 3.3 sepsis hospitalizations per 100,000 population. The adjusted *EC* of the crude sepsis incidence: *EC* = 5.9 [95% CI, 2.4, 9.4] (*p* < .001).

Among indicators of medical infrastructure, an increase of the mean distance to the nearest pharmacy by 1000 m was found to be associated with an expected increase in sepsis incidence by 21.6 [95% CI, 10.1, 33.0] sepsis hospitalizations per 100,000 inhabitants, multiple regression analyses (*p* < 0.001). A statistically significant, but weaker association was found between mean pharmacy distance and age-standardized sepsis incidence in the multiple regression analyses (Table 3, EC =  11.1 [95% CI, 1.0, 21.2], *p* = .026). All other indicators of medical capacity were not significantly associated with crude- or age-standardized sepsis incidence.

Socioeconomic indicators and health capacity indicators explained 6 and 10%, respectively, of the variance in sepsis incidence between districts in the multiple regressions including each set of indicators separately. The Pseudo-*R*^*2*^ of the full multiple NB regression with both sets of indicator variables (i.e., the indicators of socioeconomic deprivation and medical capacity, Table 3) was 0.078. Dropping one set of indicators resulted in significantly lower proportions of explained regional variation (omitting socioeconomic deprivation indicators: Δ Pseudo-*R*^*2*^ = 0.033, *p* = 0.004; omitting medical capacity indicators: Δ Pseudo-*R*^*2*^ = 0.024, *p* = 0.022). Hence, both sets of variables seem to address unique proportions of the regional variation of the sepsis incidence (supplementary file 2 Table 5). The full model with age-adjusted sepsis incidence as outcome failed statistical significance (*χ*^2^(6) = 12.27, *p* = 0.056). Accordingly, a unique proportion of regional variance was neither explained by socioeconomic deprivation nor by health care capacity.

### Comparison with implicitly identified sepsis

In comparison to hospitalizations with explicitly coded sepsis, the incidence of hospital-treated sepsis identified by implicit coding was higher (overall incidence = 1498, median = 1494 (IQR = 1306 to 1737) per 100,000 population, 6.5% of hospitalizations, supplementary file 2 Figure 8). Implicit and explicit sepsis incidence were positively correlated (*r* = 0.603). We found a similar result pattern for socioeconomic indicators as predictors for implicitly defined sepsis in the simple and multiple regression analyses, but higher Pseudo-*R*^*2*^ values indicate a stronger stochastic relationship than for the explicitly defined sepsis (Table 4). Contrary to our findings on explicit sepsis, mean household income and hospital bed capacity was positively associated with crude and age-standardized sepsis incidence in the simple and multiple regression analyses. The Pseudo-*R*^*2*^ of the full multiple NB regression including all indicators of socioeconomic deprivation and medical capacity was *R*^*2*^ = 0.209 for the crude sepsis incidence and *R*^*2*^ = 0.030 for the age-standardized sepsis incidence.

**Table 4:** Results of the simple and multiple negative binomial regression analyses for the outcome crude and age-adjusted incidence of sepsis (implicit) per 100.000 population

Predictor	Simple negative binomial regression			Multiple negative binomial regression			Multiple negative binomial regression (Full model)		
	*EC* (95% CI)	p	R^2^	*EC* (95% CI)	p	R^2^	*EC* (95% CI)	p	R^2^
**PANEL A: Outcome - crude sepsis incidence**									
Mean age	89.1 (73.2, 105.0)	< 0.001	0.242	–	–	–	–	–	–
*Socioeconomic indicators*									
Unemployment rate	42.0 (29.5, 54.5)	< 0.001	0.101	12.8 (−3.6, 29.2)	0.125	0.156	32,5 (14.0, 51.1)	< 0.001	0.209
Net household income^a,c^	−57.9 (−72.5, −43.3)	< 0.001	0.127	−36.8 (−56.7, −17.0)	< 0.001		−15,3 (− 36.5, 5.8)	0.158	
Rate of school leavers w/o certificate	49.1 (33.4, 64.9)	< 0.001	0.091	24.3 (6.9, 41.7)	0.006		17,7 (0.3, 35.0)	0.045	
*Indicators of medical infrastructure*									
Hospital beds/1000 population	10.6 (2.0, 19.3)	0.016	0.078	22.4 (8.7, 36.1)	0.001	0.138	16,7 (4.0, 29.5)	0.010	
GPs/100,000 population	0.0 (−1.3, 1.3)	0.990	0.000	−0.3 (−2.7, 2.1)	0.787		−1,4 (−3.7, 0.8)	0.213	
Distance to the next pharmacy^b^	82.5 (37.9, 127.1)	< 0.001	0.034	122.1 (63.8, 180.4)	122.1		108.0 (49.2, 166.9)	0.001	
**PANEL B: Outcome - age-adjusted sepsis incidence**									
*Socioeconomic indicators*									
Unemployment rate	20.4 (9.4, 31.4)	0.000	0.033	3.5 (−11.4, 18.3)	0.647	0.059	8.6 (−8.5, 25.6)	0.323	0.078
Net household income^a,c^	−33.8 (−47.1, −20.5)	0.000	0.056	−28.3 (−46.5, −10.1)	0.003		−20.0 (− 39.7, −0.4)	0.047	
Rate of school leavers w/o certificate	20.9 (7.1, 34.7)	0.003	0.022	5.9 (−9.8, 21.6)	0.458		4.8 (−11.3, 20.9)	0.559	
*Indicators of medical infrastructure*									
Hospital beds/1000 population	9.5 (2.0, 17.0)	0.013	0.077	18.5 (6.4, 30.6)	0.002	0.096	14.9 (3.0, 26.8)	0.013	
GPs/100,000 population	0.3 (−0.8, 1.4)	0.579	0.001	−1.0 (−3.1, 1.1)	0.352		−1.4 (− 3.5, 0.7)	0.192	
Distance to the next pharmacy^b^	26.6 (−11.1, 64.2)	0.164	0.005	43.6 (−5.5, 92.7)	0.077		29.4 (−22.9, 81.7)	0.266	

*Abbreviations*: *EC* Expected change, *w/o* without, *CI* Confidence Interval

^a^ Increase per 100 Euro

^b^ Increase per 1000 m

^c^ Simple and multiple negative binomial regression models include net household income as a predictor are based on 399 instead of 401 German districts due to missing values for two districts (Soemmerda & Fuerth)

## Discussion

In this ecological study based on complete nationwide hospital discharge data, the incidence of explicitly coded sepsis was 178 per 100,000 population in Germany and varied more than 10-fold between districts, even when adjusting for differences in age structure between districts. There were notable associations between sepsis incidence and district-level contextual factors. Our results indicate that the residence in districts with higher rates of poorly qualified school leavers and lower spatial density of medical services as mirrored by the distance to the nearest pharmacy is associated with a higher crude- and age-adjusted sepsis incidence. These associations are small, but translate to clinically meaningful increases in the number of sepsis hospitalizations. An increase in the distance to the nearest pharmacy, for example, was found to be associated with an expected increase in age-adjusted sepsis incidence of 11.1/100,000 population. Socioeconomic indicators and health capacity indicators explained 6 and 10% of the variance in sepsis incidence between districts, respectively (2 and 7% of variance in age-adjusted sepsis incidence). Considering the age differences between districts, which was found the strongest single predictor of sepsis incidence in our study, indicators of socioeconomic deprivation and health care capacity did not explain unique proportions of regional variance. This finding implies a stochastic relationship between the districts’ age distribution and the social deprivation and medical care indicators, but does not allow the conclusion that social deprivation and health care capacity do not affect the regional distribution of sepsis. Given the limited number of variables available, there are other potentially unobserved covariates at the individual- and district-level to be taken into account for estimating causal effects of social deprivation and health care capacity indicators.

The regional variation of sepsis incidence is striking and raises the question of underlying causes. Sepsis incidence is a function of both incidence of infection and the proportion of infection that progress into sepsis. Given that our analyses exclusively rely on hospital data, we cannot examine how the former, i.e. the infections, varied between German districts and contributed to the regional disparities we observed. The vulnerability for sepsis is increased in higher age groups and patients with chronic diseases or immunosuppressive therapies. The regional variation of these across German districts that was found in previous studies [15, 33, 34] may also contribute to disparities in sepsis incidence. However, common pattern of regional health disparities in Germany, e.g. higher incidence of cardiovascular diseases [35], cardiovascular risk factors [36] and diabetes [37] observed in the federal states of former Eastern Germany compared to Western Germany, were not evident in our analyses. Variation can therefore also reflect differences in sepsis awareness and diagnosis, which may be increased by local and regional sepsis quality improvement or sepsis awareness programs [38, 39], e.g. conducted in Thuringia and Mecklenburg Western Pomerania, impacting the number of sepsis cases diagnosed and coded by ICD-10-GM codes in administrative data.

We found that the proportion of school leavers without certificate was the only indicator for socioeconomic deprivation associated with increased crude and age-standardized sepsis incidence rates. Although described for other diseases in Germany such as hypertension, obesity [40] or diabetes [14], associations between other socioeconomic indicators such household income or unemployment rate and sepsis incidence were not provable in our data. The educational level is constrained by educational opportunities in a society and family background and is associated with income and occupational position. It is thus considered as a meaningful indicator for socioeconomic status [22, 41]. Given that association between such socioeconomic status and the incidence of infection may arise from complex links between environmental exposures, access to transportation, and care, e.g. for the management of chronic conditions, and health risk behaviours [42] as well as health status and disease in general [21], we lack understanding why we found no such effects for the indicators of income or unemployment. A more complete picture of the (causal) effects of individual and context socioeconomic factors on sepsis incidence requires more evidence from patient-level cohort studies.

Another novel finding of our study is that the outpatient and inpatient capacity as expressed by the number of GPs and hospital beds per population were not significantly associated with the incidence of explicit sepsis in Germany. However, there was a positive association between spatial distance to the nearest pharmacy as surrogate for density of medical services, and sepsis incidence, which to our knowledge has not been described before. Longer distances to medical services can particularly affect elderly patients in rural areas with limited mobility [43] and can pose a major barrier to the health care of patients with chronic diseases [44, 45], thus increasing the risk for sepsis by delays or inappropriate antibiotic treatment of infections that may cause sepsis [46]. We chose the distance to the next pharmacy rather than urbanity as indicator since previous studies have shown that the urbanity and accessibility of inpatient health care are only correlated by *r* = 0.31 [47]. However, the extent to which the proximity to pharmacies matches with the distance of other medical services including hospitals, which are crucial for the treatment of sepsis patients, remains unknown. A positive association between GP and pharmacy accessibility was reported from the United Kingdom [48]. A 1% increase in overall community pharmacy access corresponded to a 0.86% increase in GP access, with a higher gradient found in urban compared to rural areas [48].

We compared associations between contextual factors and implicitly and explicitly defined sepsis as explicit coding might be influenced by sepsis awareness of health care professionals in the treating hospitals and monetary incentives in the DRG system [49]. Generally, explicit coding strategies were found to underestimate the burden of clinically defined sepsis [50, 51], while implicit coding strategies lead to an overestimation of incidence rates [50]. Explicit and implicit sepsis incidence was correlated by *r* = 0.603 in our study and the incidence of implicit sepsis was more than 8-fold higher compared to the incidence of explicit sepsis, which is approximately twice the difference observed in previous US-studies [51]. Undercoding of explicit sepsis codes due to poor sepsis awareness is a potential explanation for the lower stochastic dependencies between predictors and incidence rates of explicitly compared to implicitly defined sepsis. This may also explain the positive association between implicit sepsis incidence and mean household income, and sepsis incidence and hospital bed capacity in the simple and multiple regression analyses, which were not found for explicitly defined sepsis. The underlying mechanism of the associations between the hospital bed capacity and the occurrence of sepsis are still unclear.

The following limitations of our study need to be considered. First, this is an ecological study, thus the observed associations cannot be interpreted as causal. Second, unbiased parameter estimates depend upon the validity of sepsis coding in hospital discharge data, which was found to be limited in a single center validation study [50] and may vary between hospitals. Third, as the DRG statistics are anonymized hospital episode statistics, our analyses were limited to hospitalizations, not individual patients. Thus, we were unable to identify hospital transfers and multiple sepsis episodes in one patient, which in case of varying transfer practices can impact the district-level sepsis incidence estimates. Fourth, we did investigate one selected year, thus it remains unknown if these findings can be replicated with data of other years. Context factors itself as well as their correlational structure may change over time. Hence, we cannot make any conclusions about temporal trends in associations between sepsis incidence and context factors. Further studies are needed to close these gaps.

## Conclusions

Lower district-level socioeconomic status (e.g., less education) and proximity of medical services were found to be associated with an increased sepsis incidence, while the ratio of hospital beds and GPs were not similarly associated with sepsis incidence. Further cohort studies are required to investigate the regional context factors as potential risk factors for sepsis at the individual patient level. Subsequent identification of causal factors behind the ecological relations observed in this study can inform future interventions to reduce the sepsis incidence.

## Supplementary Information

**Additional file 1.** Supplement to Methods/Definitions. Supplementary file 1 includes case and indicator definitions.

**Additional file 2.** Supplementary Figures and Tables. Supplementary file 2 includes supplemental Figures and Tables.

**Additional file 3.** Supplement to Methods/Statistical analyses. Supplementary file 3 includes a supplementary statistical analyses section.

## Data Availability

The data of the DRG statistics, which is used in this study, was kindly provided by the Federal Statistical Office and was analysed by remote data processing. To use the data, a data use contract has to be established with the Federal Statistical Office. Thus, only the Federal Statistical Office can grant access to the data.

## Abbreviations

DRG: Diagnosis-related Groups
EC: Expected change
EPC: Expected percentage change
GP: General practitioner
ICC: Intraclass correlation
ICD-10-GM: International Classification of Diseases, Tenth Revision, German Modification
INKAR: Indicators and maps on spatial and urban development
IQR: Interquartile range
NB: Negative binomial
US: United States
w/o: without
