# Supplementary material for: Association between sepsis incidence and regional socioeconomic deprivation and health care capacity in Germany – an ecological study
Source: BMC Public Health. 2021 Sep 7;21:1636. doi: 10.1186/s12889-021-11629-4 (PMC8424852; doi:10.1186/s12889-021-11629-4)
Supplement: Supplementary file 1 — Additional file 1. Supplement to Methods/Definitions. Supplementary file 1 includes case and indicator definitions. [file 12889_2021_11629_MOESM1_ESM.docx]

Supplementary file 1

**Association between sepsis incidence and regional socioeconomic deprivation and health care capacity in Germany – An ecological study**

Dr. Norman Rose^1,2^, Dr. Claudia Matthäus-Krämer^1^, Dr. Daniel Schwarzkopf^2,3^, Prof. André Scherag^4^, Dr. Sebastian Born^1,2^, Prof. Konrad Reinhart^5^, Dr. Carolin Fleischmann-Struzek^1,2^

1 Center for Sepsis Control and Care, Jena University Hospital, Bachstraße 18, 07743 Jena, Germany

2 Institute of Infectious Diseases and Infection Control, Jena University Hospital, Am Klinikum 1, 07747 Jena, Germany

3 Department for Anesthesiology and Intensive Care Medicine, Jena University Hospital,

Am Klinikum 1, 07740 Jena, Germany,

4 Institute of Medical Statistics, Computer and Data Sciences, Jena University Hospital, Bachstraße 18, 07743 Jena, Germany

5 Department of Anesthesiology and Intensive Care Medicine, Charité Universitätsmedizin Berlin, Charitéplatz 1, 10117 Berlin, Germany

**ICD-10-GM definitions**

Explicit sepsis

R65.1 - Systemic Inflammatory Response Syndrome of infectious origin with organ failure, R57.2 - Septic shock

Implicit sepsis

ICD-10-GM codes for infection PLUS organ dysfunction

ICD-10-GM infection codes: J01 - Acute sinusitis, J02 - Acute pharyngitis, J03 - Acute tonsillitis, J04 - Acute laryngitis and tracheitis, J06 - Acute upper respiratory infections of multiple and unspecified sites, J05 - Acute obstructive laryngitis [croup] and epiglottitis, J09 - Influenza due to identified zoonotic or pandemic influenza virus, J10 - Influenza due to identified seasonal influenza virus, J11 - Influenza, virus not identified, J12 - Viral pneumonia, not elsewhere classified, J13 - Pneumonia due to Streptococcus pneumoniae, J14 - Pneumonia due to Haemophilus influenzae, J15 - Bacterial pneumonia, not elsewhere classified, J16 - Pneumonia due to other infectious organisms, not elsewhere classified, J17 - Pneumonia in diseases classified elsewhere, J18 - Pneumonia, organism unspecified, J20 - Acute bronchitis, J21 - Acute bronchiolitis, J22 - Unspecified acute lower respiratory infection, J44.0 - Chronic obstructive pulmonary disease with acute lower respiratory infection, J44.1 - Chronic obstructive pulmonary disease with acute exacerbation, unspecified, J86 - Pyothorax, J85 - Abscess of lung and mediastinum, A15 - Respiratory tuberculosis, bacteriologicallyorhistologically confirmed, A16 - Respiratory tuberculosis, not confirmed bacteriologically or histologically, U69.00 - Hospital-acquired pneumonia in other diseases classified elsewhere, A36 - Diphtheria, A37 - Whooping cough, B38 - Coccidioidomycosis, B39 - Histoplasmosis

A00 - Cholera, A01 - Typhoid and paratyphoid fevers, A02 - Other salmonella infections, A03 - Shigellosis, A04 - Other bacterial intestinal infections, A05 - Other bacterial foodborne intoxications, not elsewhere classified, A06 - Amoebiasis, A07 - Other protozoal intestinal diseases, A08 - Viral and other specified intestinal infections, A09 - Other gastroenteritis and colitis of infectious and unspecified origin, K35 - Acute appendicitis, K37 - Unspecified appendicitis, K36 - Other appendicitis, K5702 - Diverticular disease of small intestine with perforation and abscess without bleeding, K5703 - Diverticular disease of small intestine with perforation and abscess with bleeding, K5712 - Diverticular disease of small intestine without perforation or abscess without bleeding, K57.13 - Diverticular disease of small intestine without perforation or abscess wit bleeding, K57.22 - Diverticular disease of large intestine with perforation and abscess without bleeding, K57.23 - Diverticular disease of large intestine with perforation, abscess and bleeding, K57.32 - Diverticular disease of large intestine without perforation or abscess wihout bleeding, K5733 - Diverticular disease of large intestine without perforation or abscess wit bleeding, K5742 - Diverticular disease of both small and large intestine with perforation and abscess without bleeding, K5743 - Diverticular disease of both small and large intestine with perforation, abscess and bleeding, K5752 - Diverticular disease of both small and large intestine without perforation or abscess or bleeding, K5753 - Diverticular disease of both small and large intestine without perforation or abscess with bleeding, K5782 - Diverticular disease of intestine, part unspecified, with perforation and abscess without bleeding, K5783 - Diverticular disease of intestine, part unspecified, with perforation, abscess and bleeding, K5792 - Diverticular disease of intestine, part unspecified, without perforation, abscess or bleeding, K5793 - Diverticular disease of intestine, part unspecified, without perforation or abscess with bleeding, K61 - Abscess of anal and rectal regions, K65 - Peritonitis, K67 - Disorders of peritoneum in infectious diseases classified elsewhere, K63.0 - Abscess of intestine, K63.1 - Perforation of intestine (nontraumatic), K75.0 - Abscess of liver, K75.1 - Phlebitis of portal vein, K81.0 - Cholecystitis, K77.0 - Liver disorders in infectious and parasitic diseases classified elsewhere, U69.40! - Recurrent infection due to Clostridium difficile

A46 - Erysipelas, B47 - Mycetoma, L03 - Phlegmon, L04 - Acute lymphadenitis, L08 - Other local infections of skin and subcutaneous tissue, L05 - Pilonidal cyst, B00 - Herpesviral [herpes simplex] infections, B07 - Viral warts, B08 - Other viral infections characterized by skin and mucous membrane lesions, not elsewhere classified, B09 - Unspecified viral infection characterized by skin and mucous membrane lesions, H05.0 - Acute inflammation of orbit, H60.2 - Malignant otitis externa, H70.0 - Acute mastoiditis, J36 - Peritonsillar abscess, J39.0 - Retropharyngeal and parapharyngeal abscess, J39.1 - Other abscess of pharynx, L02 - Cutaneous abscess, furuncle and carbuncle

N10 - Acute tubulo-interstitial nephritis, N15.1 - Renal and perinephric abscess, N15.9 - Renal tubulo-interstitial disease, unspecified, N34 - Urethritis and urethral syndrome, N30 - Cystitis, N39.0 - Urinary tract infection, site not specified, N41 - Inflammatory diseases of prostate, N45 - Orchitis and epididymitis, N48.2 - Other inflammatory disorders of penis, N49 - Inflammatory disorders of male genital organs, not elsewhere classified, N70 - Salpingitis and oophoritis, N71 - Inflammatory disease of uterus, except cervix, N72 - Inflammatory disease of cervix uteri, N73 - Other female pelvic inflammatory diseases, N74 - Female pelvic inflammatory disorders in diseases classified elsewhere, N75 - Diseases of Bartholin gland, N76 - Other inflammation of vagina and vulva, N77 - Vulvovaginal ulceration and inflammation in diseases classified elsewhere, N61 - Inflammatory disorders of breast, N98.0 - Infection associated with artificial insemination, A59 - Trichomoniasis, A55 - Chlamydial lymphogranuloma (venereum), A56 - Other sexually transmitted chlamydial diseases

A39 - Meningococcal infection, G00 - Bacterial meningitis, not elsewhere classified, G01 - Meningitis in bacterial diseases classified elsewhere, G02 - Meningitis in other infectious and parasitic diseases classified elsewhere, G03 - Meningitis due to other and unspecified causes, G04 - Encephalitis, myelitis and encephalomyelitis, G05* - Encephalitis, myelitis and encephalomyelitis in diseases classified elsewhere, G06 - Intracranial and intraspinal abscess and granuloma, G07* - Intracranial and intraspinal abscess and granuloma in diseases classified elsewhere, G08 - Intracranial and intraspinal phlebitis and thrombophlebitis, A17+ - Tuberculosis of nervous system, A81 - Atypical virus infections of central nervous system, A83 - Mosquito-borne viral encephalitis, A84 - Tick-borne viral encephalitis, A85 - Other viral encephalitis, not elsewhere classified, A86 - Unspecified viral encephalitis, A87 - Viral meningitis, A88 - Other viral infections of central nervous system, not elsewhere classified, A89 - Unspecified viral infection of central nervous system

I32 - Pericarditis in diseases classified elsewhere, I33 - Acute and subacute endocarditis, I39 - Endocarditis and heart valve disorders in diseases classified elsewhere, I40 - Acute myocarditis, I41 - Myocarditis in diseases classified elsewhere, I80 - Thombosis, phlebitis and thrombophlebitis, I38 - Endocarditis, valve unspecified, I98.1 - Cardiovascular disorders in other infectious and parasitic diseases classified elsewhere

T82.6 - Infection and inflammatory reaction due to cardiac valve prosthesis, T82.7 - Infection and inflammatory reaction due to other cardiac and vascular devices , implants and grafts, T83.5 - Infection and inflammatory reaction due to prosthetic device, implant and graft in urinary system, T83.6 - Infection and inflammatory reaction due to prosthetic device, implant and graft in genital tract, T84.5 - Infection and inflammatory reaction due to internal joint prosthesis, T84.6 - Infection and inflammatory reaction due to internal fixation device [any site], T84.7 - Infection and inflammatory reaction due to other internal orthopaedic prosthetic devices, implants and grafts, T85.7 - Infection and inflammatory reaction due to other internal prosthetic devices, implants and grafts

O75.3 - Other infection during labour, O85 - Puerperal fever, O030 - Spontaneous abortion; Incomplete, complicated by genital tract and pelvic infection, O035 - Spontaneous abortion; Complete or unspecified, complicated by genital tract and pelvic infection, O040 - Medical abortion; Incomplete, complicated by genital tract and pelvic infection, O045 - Medical abortion; Complete or unspecified, complicated by genital tract and pelvic infection, O050 - Other abortion; Incomplete, complicated by genital tract and pelvic infection, O055 - Other abortion; Complete or unspecified, complicated by genital tract and pelvic infection, O060 - unspecified abortion; Incomplete, complicated by genital tract and pelvic infection, O065 - Unspecified abortion; Complete or unspecified, complicated by genital tract and pelvic infection, O070--O07.5 - Failed medical abortion, complicated by genital tract and pelvic infection, O075 - Other and unspecified failed attempted abortion, complicated by genital tract and pelvic infection, O08.0 - Genital tract and pelvic infection following abortion and ectopic and molar pregnancy, O86 - Other puerperal infections, O23 - Infections of genitourinary tract in pregnancy, O41.1 - Infection of amniotic sac and membranes, O88.3 - Obstetric pyaemic and septic embolism, O91 - Infections of breast associated with childbirth, O98 - Maternal infectious and parasitic diseases classifiable elsewhere but complicating pregnancy, childbirth and the puerperium,

Organ dysfunction

Cardiovascular dysfunction/shock

I95.9 - Hypotension, unspecified, R57.8 - Other shock, R57.9 - Shock, unspecified, R57.2 - Septic shock

Respiratory dysfunction

J96. - Respiratory failure, not elsewhere classified, J80 - Adult respiratory distress syndrome, J98.4 - Other disorders of lung, R06.0 - Dyspnoea, R06.8 - Other and unspecified abnormalities of breathing

Encephalopathy

F05 - Delirium, not induced by alcohol and other psychoactive substances, G93.1 - Anoxic brain damage, not elsewhere classified, G93.4 - Encephalopathy, unspecified, R40 - Somnolence, stupor and coma

Renal dysfunction

N17. - Acute renal failure, N19 - Unspecified kidney failure,

Metabolic dysfunction

E87.2 - Acidosis

Abnormal coagulation

Coagulation D65 - Disseminated intravascular coagulation [defibrination syndrome], D68.8 - Other specified coagulation defects, D68.9 - Coagulation defect, unspecified, D69.5 - Secondary thrombocytopenia, D69.6 - Thrombocytopenia, unspecified,

Hepatic dysfunction

K72.0 Acute and subacute hepatic failure, K72.7 - Hepatic encephalopathy and hepatic coma, K72.9 - Hepatic failure, unspecified, K76.2 - Central haemorrhagic necrosis of liver, K76.3 - Infarction of liver,

Other organ dysfunction

R65.1 - Systemic Inflammatory Response Syndrome of infectious origin with organ complications

**Definition of the airline distance to the nearest pharmacy**

The airline distance to the nearest pharmacy is the average of the distances of individuals’ home to the next pharmacy within a district. However, the mean is difficult to be computed directly. Therefore, this quantity is approximated by partitioning each district into 250 x 250m spatial units (*SU*). The distances *D_i_* between each *SU_i_* to the next *SU_j_* with a pharmacy is computed. Then a weighted mean of the distances *D_i_* is computed. The weights are based on the proportions of inhabitants in the *SUs*.
