# Supplementary material for: Association between sepsis incidence and regional socioeconomic deprivation and health care capacity in Germany – an ecological study
Source: BMC Public Health. 2021 Sep 7;21:1636. doi: 10.1186/s12889-021-11629-4 (PMC8424852; doi:10.1186/s12889-021-11629-4)
Supplement: Supplementary file 2 — Additional file 2. Supplementary Figures and Tables. Supplementary file 2 includes supplemental Figures and Tables. [file 12889_2021_11629_MOESM2_ESM.docx]

Supplementary file 2

**Association between sepsis incidence and regional socioeconomic deprivation and health care capacity in Germany – An ecological study**

Dr. Norman Rose^1,2^, Dr. Claudia Matthäus-Krämer^1^, Dr. Daniel Schwarzkopf^2,3^, Prof. André Scherag^4^, Dr. Sebastian Born^1,2^, Prof. Konrad Reinhart^5^, Dr. Carolin Fleischmann-Struzek^1,2^

1 Center for Sepsis Control and Care, Jena University Hospital, Bachstraße 18, 07743 Jena, Germany

2 Institute of Infectious Diseases and Infection Control, Jena University Hospital, Am Klinikum 1, 07747 Jena, Germany

3 Department for Anesthesiology and Intensive Care Medicine, Jena University Hospital,

Am Klinikum 1, 07740 Jena, Germany,

4 Institute of Medical Statistics, Computer and Data Sciences, Jena University Hospital, Bachstraße 18, 07743 Jena, Germany

5 Department of Anesthesiology and Intensive Care Medicine, Charité Universitätsmedizin Berlin, Charitéplatz 1, 10117 Berlin, Germany

**Figures**

Maps were created using the ‘spplot’ function from the ‘sp’ package [1, 2]. Geodata and shapefiles for creating maps of Germany in R were retrieved from https://gadm.org/. The maps are freely available for academic use.

**Figure 1: Distribution of mean population age across the 401 German districts**

**­­­­
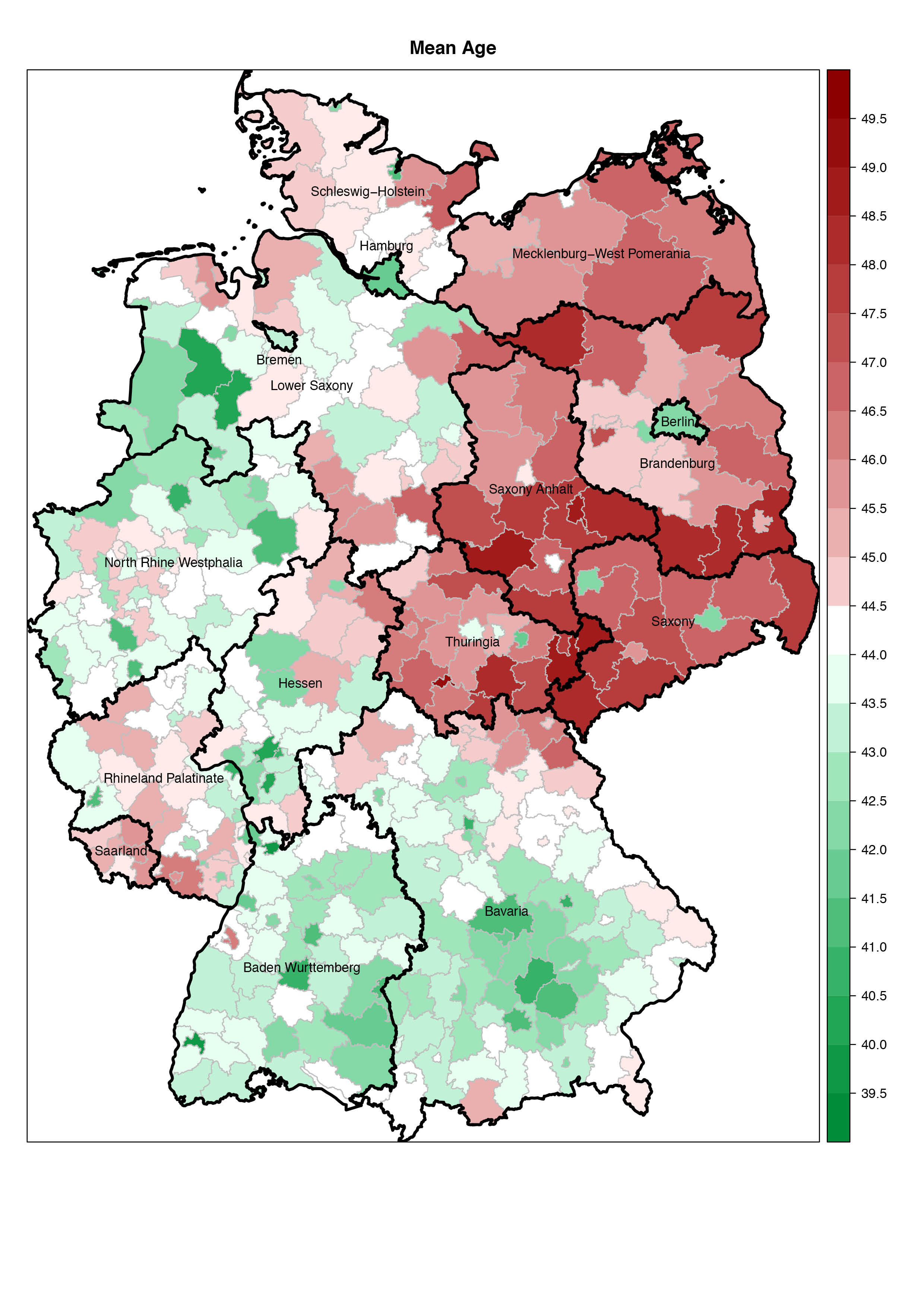
**

**Figure 2: Distribution of the unemployment rate across the 401 German districts**

**
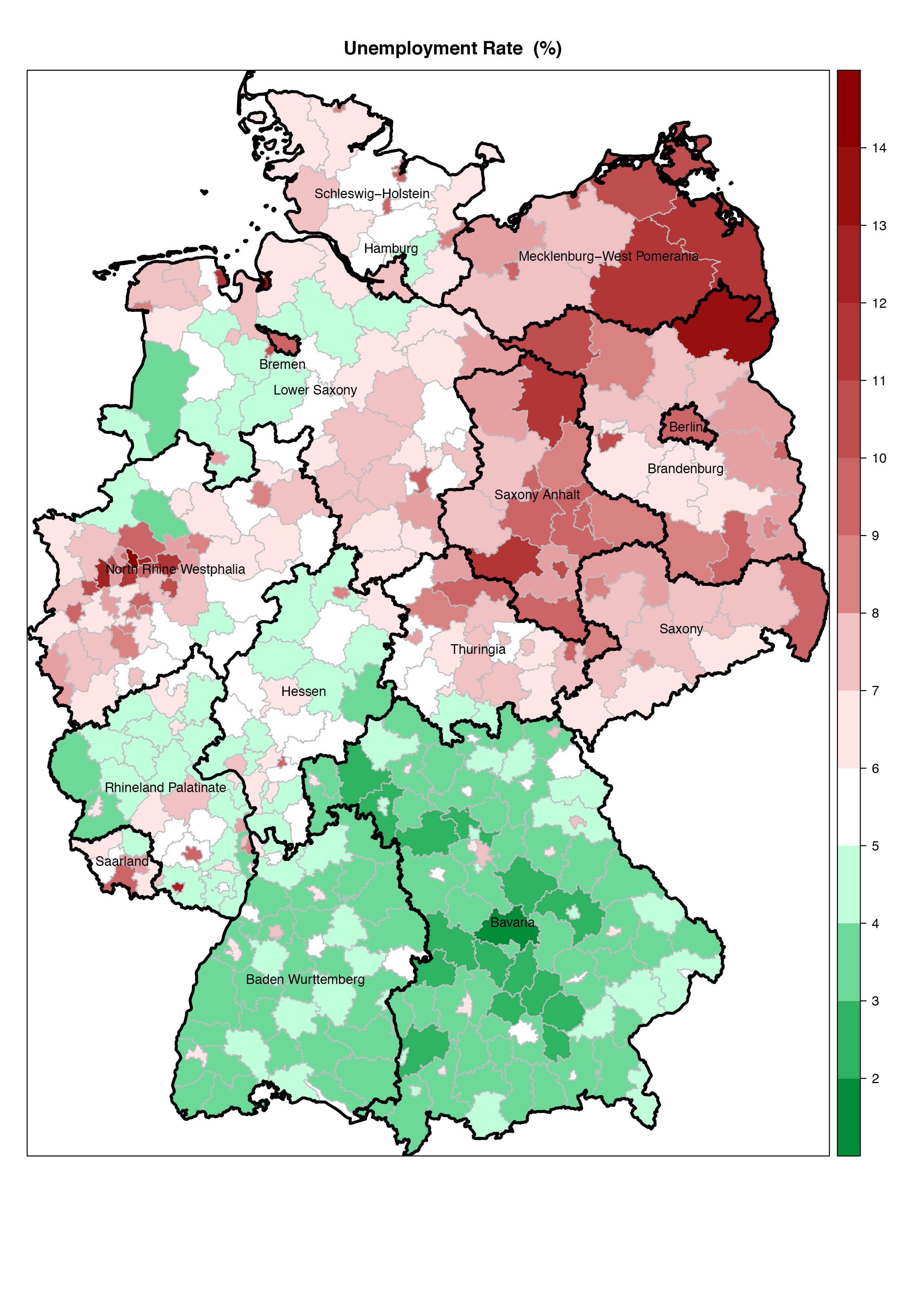
**

**Figure 3: Distribution of the net household income across the 401 German districts**

**
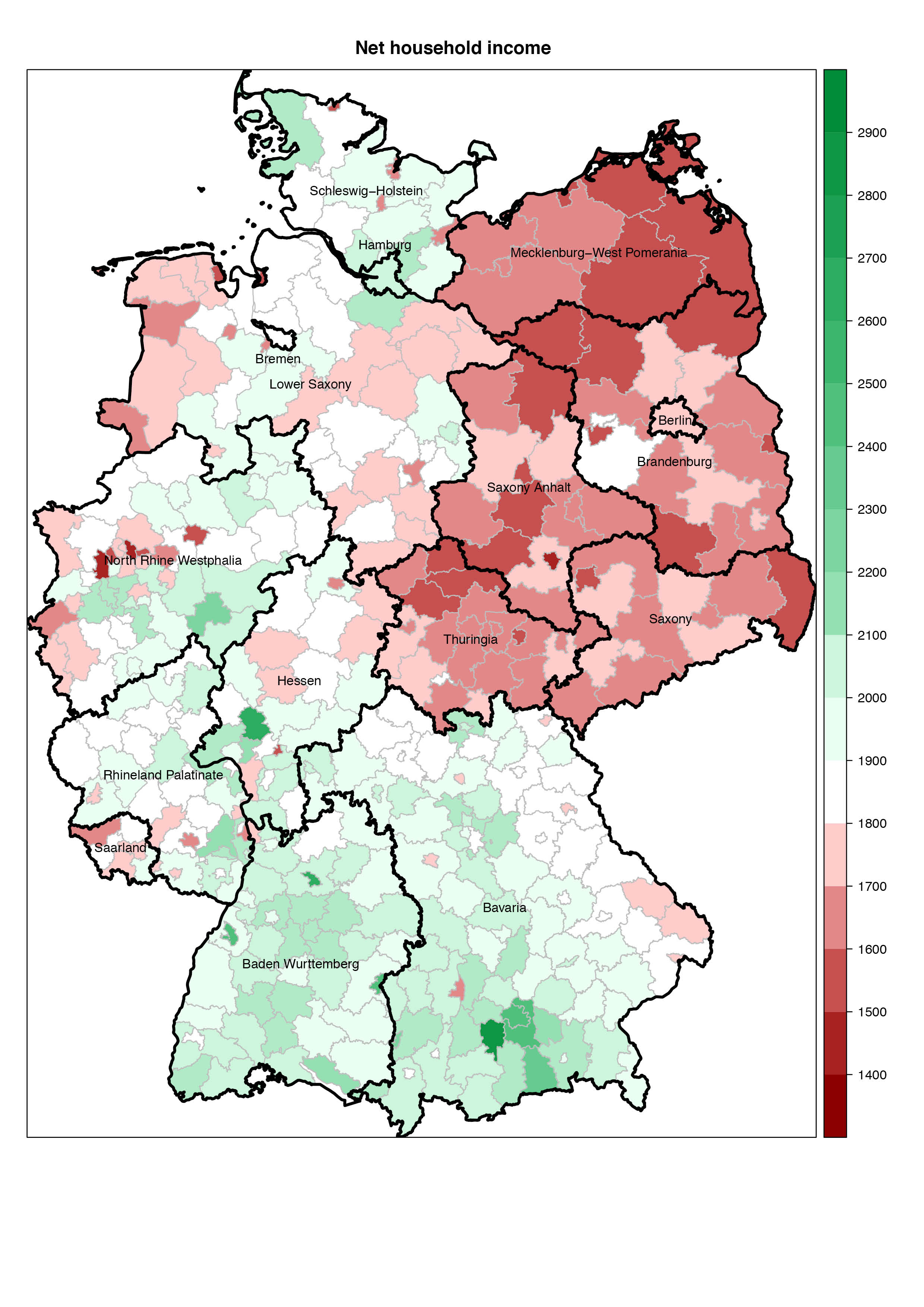
**

**Figure 4: Distribution of the rate of school leavers w/o certificate across the 401 German districts**

**
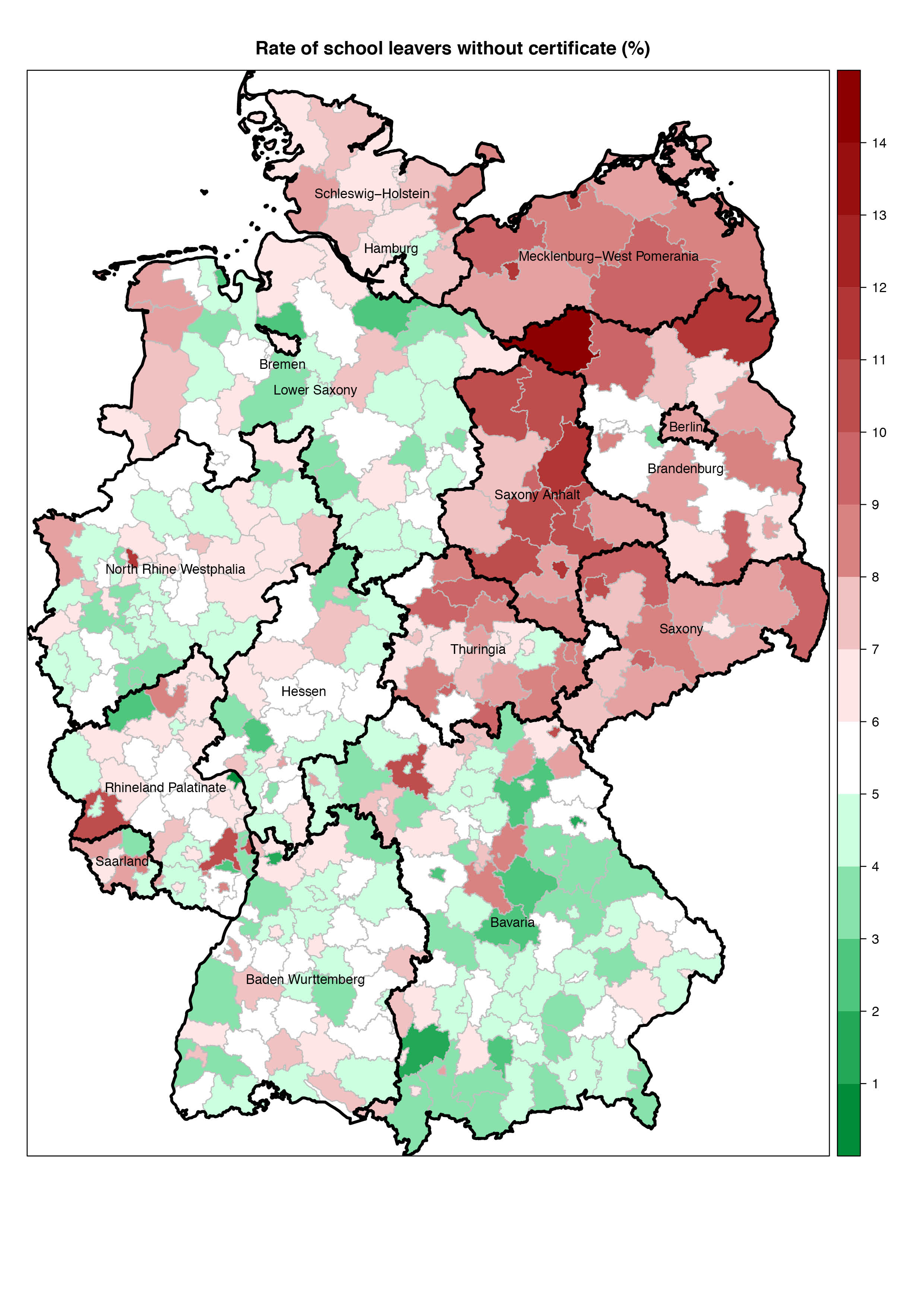
**

**Figure 5: Distribution of hospital beds/1000 population** **across the 401 German districts**

**
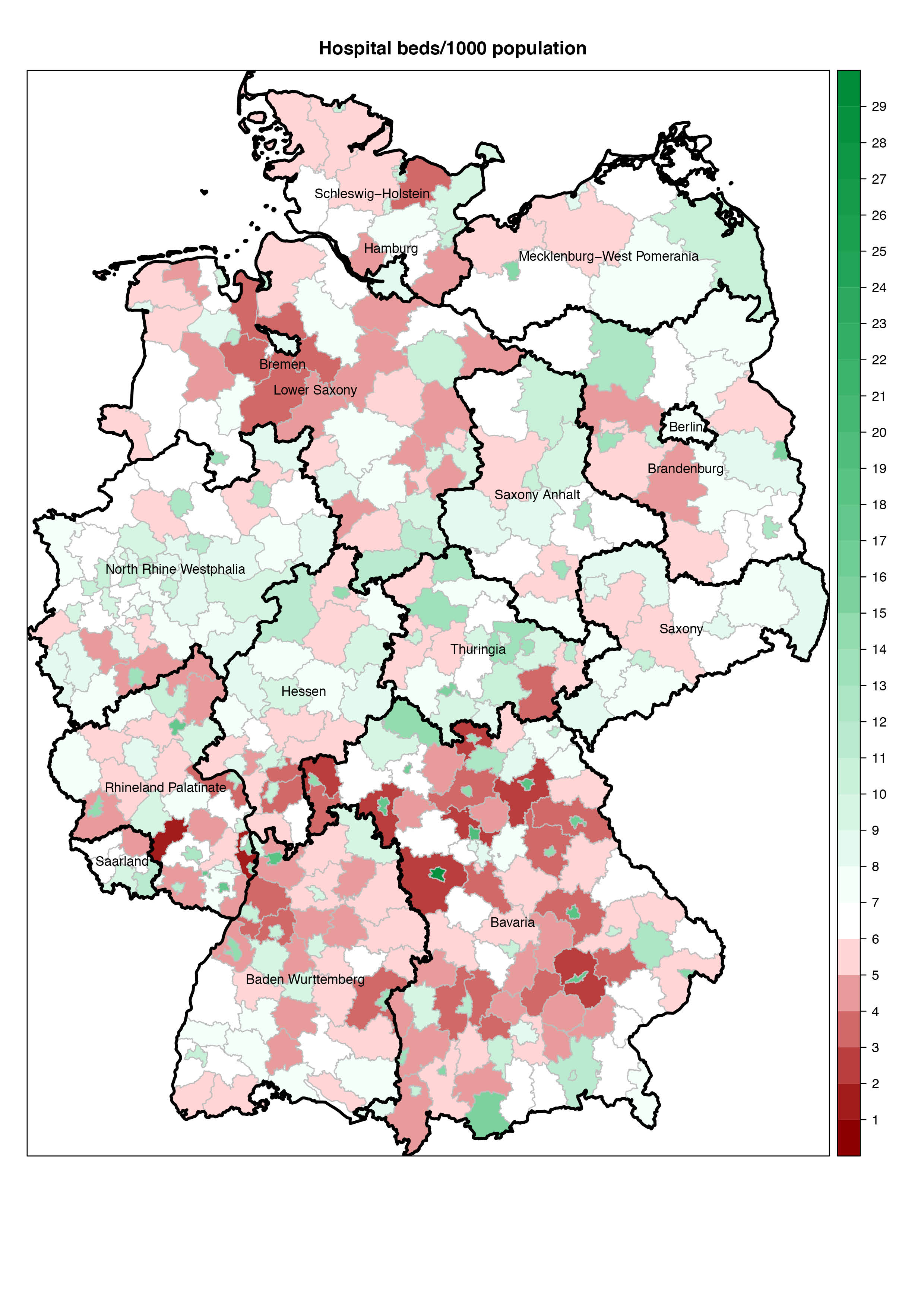
**

**Figure 6: Distribution of general practitioners/1000 population** **across the 401 German districts**

**
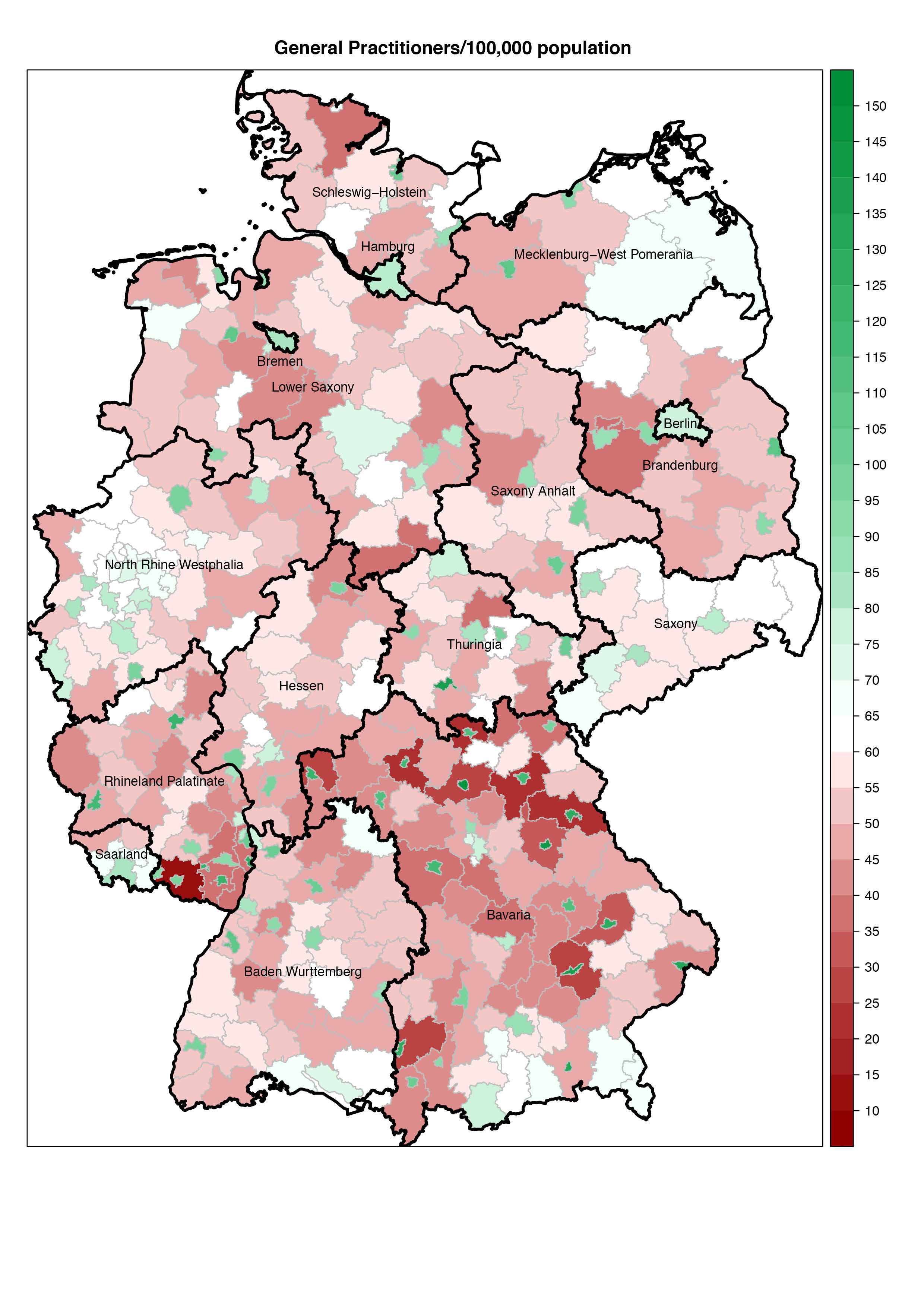
**

**Figure 7: Distribution of the distance to the next pharmacy across the 401 German districts**

**
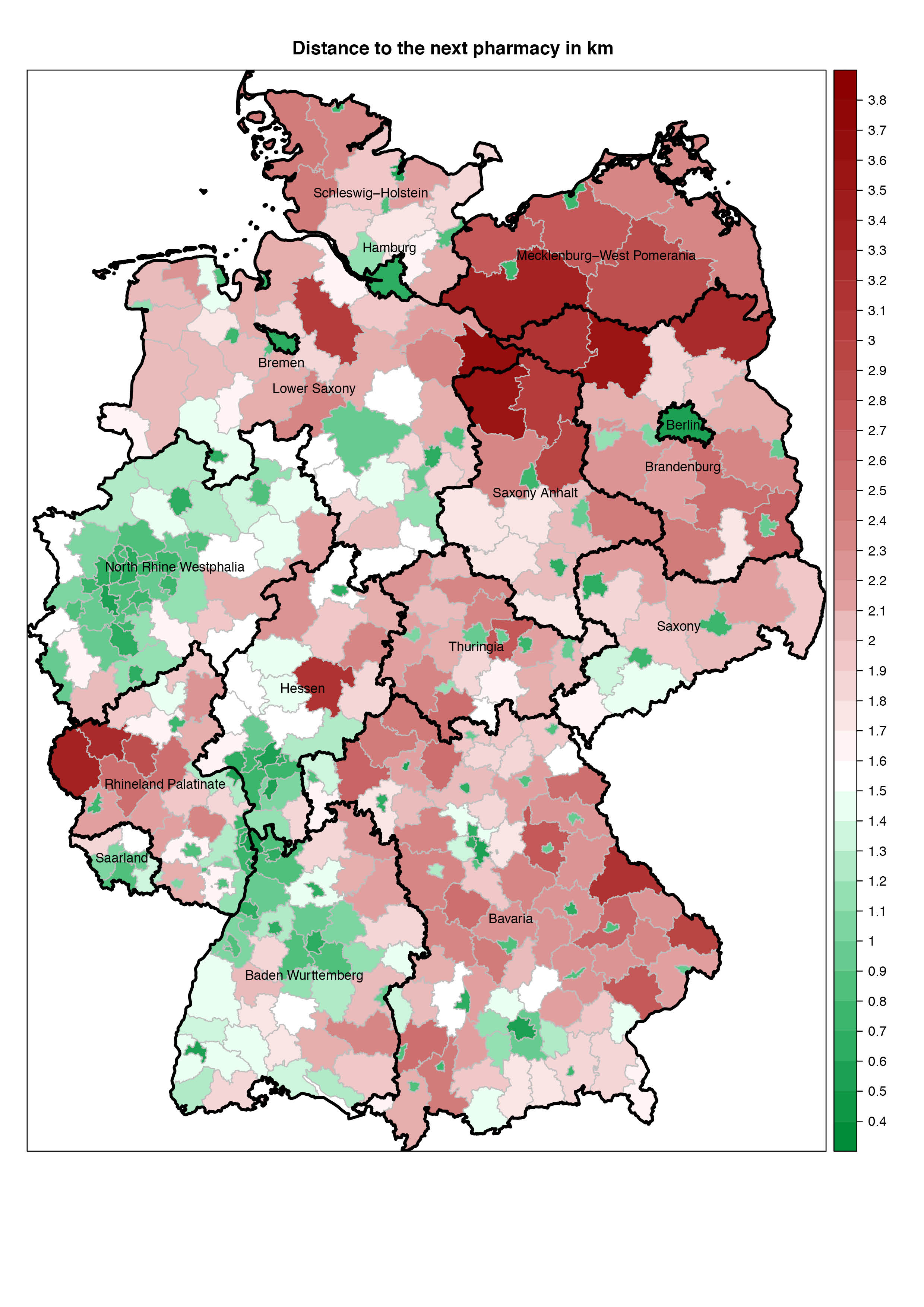
**

**Figure 8: Distribution of implicit sepsis incidence across the 401 German districts**

**
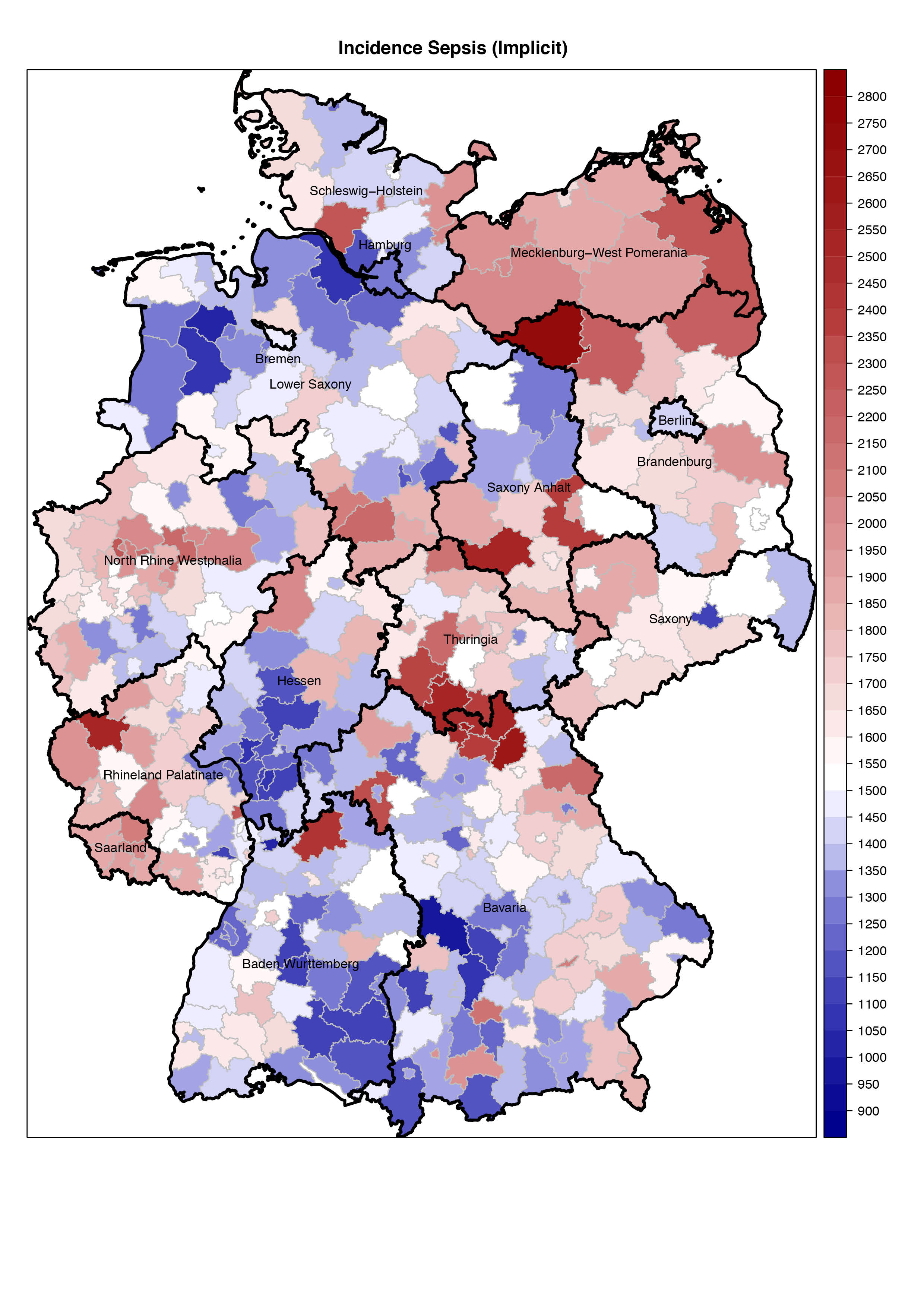
**

**Tables**

**Table 1: Regression coefficients of the negative binomial regression model, the expected percentage change (*EPC_j_*), the dispersion parameter (*θ*), and the *χ*^2^-Test (i.e., the likelihood ratio test) for overdispersion. Outcomes: Sepsis incidence rates.**

| **Outcome: Incidence of Sepsis (explicit)** | | | | | | | | | | | |
| --- | --- | --- | --- | --- | --- | --- | --- | --- | --- | --- | --- |
| **Model** | **Predictor/Intercept** | *β* | *SE* | *p* | *EPC_j_* | 95% CI | | *θ* | *SE* | *χ*^2^ | *p* |
| simple NB | Intercept | -9.23895 | 0.36526 | <0.001 |  |  |  | 10.31 | 0.75 | 10727.9 | <0.001 |
|  | Mean age | 0.06619 | 0.00821 | <0.001 | 6,843 | 5,132 | 8,590 |  |  |  |  |
| simple NB | Intercept | -6.36740 | 0.04144 | <0.001 |  |  |  | 8.94 | 0.65 | 11920.9 | <0.001 |
|  | Unemployment rate | 0.01346 | 0.00654 | 0.040 | 1.355 | 0.076 | 2.661 |  |  |  |  |
| simple NB | Intercept | -5.78590 | 0.14936 | <0.001 |  |  |  | 9.12 | 0.66 | 11645.4 | <0.001 |
|  | Net household income (100 Euro) | -0.02794 | 0.00821 | 0.001 | -2.755 | -4.234 | -1.237 |  |  |  |  |
| simple NB | Intercept | -6.49923 | 0.05064 | <0.001 |  |  |  | 9.28 | 0.67 | 11423.4 | <0.001 |
|  | Rate of school leavers w/o certificate | 0.03474 | 0.00800 | <0.001 | 3.535 | 1.951 | 5.157 |  |  |  |  |
| multiple NB | Intercept | -6.01409 | 0.24654 | <0.001 |  |  |  | 9.38 | 0.65 | 11212.9 | <0.001 |
|  | Unemployment rate | -0.01145 | 0.00886 | 0.196 | -1.139 | -2.820 | 0.586 |  |  |  |  |
|  | Net household income (100 Euro) | -0.02212 | 0.01106 | 0.046 | -2.187 | -4.174 | -0.117 |  |  |  |  |
|  | Rate of school leavers w/o certificate | 0.03140 | 0.00934 | 0.001 | 3.190 | 1.325 | 5.100 |  |  |  |  |
| simple NB | Intercept | -6.33138 | 0.03311 | <0.001 |  |  |  | 8.86 | 0.64 | 12395.4 | <0.001 |
|  | Hospital beds/1000 population | 0.00654 | 0.00446 | 0.142 | 0.656 | -0.236 | 1.569 |  |  |  |  |
| simple NB | Intercept | -6.29463 | 0.04430 | <0.001 |  |  |  | 8.84 | 0.64 | 12523.7 | <0.001 |
|  | GPs/100,000 population | 0.00009 | 0.00068 | 0.891 | 0.009 | -0.122 | 0.142 |  |  |  |  |
| simple NB | Intercept | -6.39328 | 0.03688 | <0.001 |  |  |  | 9.06 | 0.66 | 12077.4 | <0.001 |
|  | Distance to the next pharmacy (1000 m) | 0.06853 | 0.02183 | 0.002 | 7.094 | 2.556 | 11.849 |  |  |  |  |
| multiple NB | Intercept | -6.58398 | 0.08761 | <0.001 |  |  |  | 9.26 | 0.67 | 11613.9 | <0.001 |
|  | Hospital beds/1000 population | 0.01097 | 0.00706 | 0.120 | 1.103 | -0.320 | 2.565 |  |  |  |  |
|  | GPs/100,000 population | 0.00094 | 0.00126 | 0.456 | 0.094 | -0.152 | 0.341 |  |  |  |  |
|  | Distance to the next pharmacy (1000 m) | 0.11020 | 0.02817 | <0.001 | 11.650 | 5.527 | 18.171 |  |  |  |  |
| Multiple NB (full model) | Intercept | -6.49011 | 0.28943 | <0.001 |  |  |  | 9.58 | 0.67 | 10633.9 | <0.001 |
|  | Unemployment rate | -0.00142 | 0.01007 | 0.888 | -0.142 | -2.072 | 1.842 |  |  |  |  |
|  | Net household income (100 Euro) | -0.00844 | 0.01187 | 0.477 | -0.841 | -3.049 | 1.465 |  |  |  |  |
|  | Rate of school leavers w/o certificate | 0.02629 | 0.00955 | 0.006 | 2.663 | 0.774 | 4.598 |  |  |  |  |
|  | Hospital beds/1000 population | 0.00978 | 0.00703 | 0.164 | 0.983 | -0.422 | 2.425 |  |  |  |  |
|  | GPs/100,000 population | 0.00023 | 0.00126 | 0.858 | 0.022 | -0.221 | 0.268 |  |  |  |  |
|  | Distance to the next pharmacy (1000m) | 0.08281 | 0.03054 | 0.007 | 8.634 | 2.157 | 15.570 |  |  |  |  |
| **Outcome: Incidence of Sepsis (implicit)** | | | | | | | | | | | |
| **Model** | **Predictor/Intercept** | *β* | *SE* | *p* | *EPC_j_* | 95% CI | | *θ* | *SE* | *χ*^2^ | *p* |
| simple NB | Intercept | -6,67784 | 0,22092 | <0.001 |  |  |  | 27,46 | 1,96 | 36501,5 | < 0.001 |
|  | Mean age | 0,05632 | 0,00497 | <0.001 | 5.793 | 4.760 | 6..838 |  |  |  |  |
| simple NB | Intercept | -4.32643 | 0.02544 | <0.001 |  |  |  | 23.17 | 1.65 | 41607.3 | < 0.001 |
|  | Unemployment rate | 0.02685 | 0.00402 | <0.001 | 2.721 | 1.913 | 3.540 |  |  |  |  |
| simple NB | Intercept | -3.48057 | 0.09118 | <0.001 |  |  |  | 23.86 | 1.70 | 40675.0 | <0.001 |
|  | Net household income (100 Euro) | -0.03827 | 0.00501 | <0.001 | -3.755 | -4.678 | -2.816 |  |  |  |  |
| simple NB | Intercept | -4.35837 | 0.03181 | <0.001 |  |  |  | 22.90 | 1.63 | 44711.5 | <0.001 |
|  | Rate of school leavers w/o certificate | 0.03134 | 0.00503 | <0.001 | 3.184 | 2.181 | 4.202 |  |  |  |  |
| multiple NB | Intercept | -3.87708 | 0.14984 | <0.001 |  |  |  | 24.67 | 1.76 | 39417.5 | <0.001 |
|  | Unemployment rate | 0.00826 | 0.00539 | 0.125 | 0.829 | -0.218 | 1.893 |  |  |  |  |
|  | Net household income (100 Euro) | -0.02418 | 0.00673 | <0.001 | -2.389 | -3.642 | -1.104 |  |  |  |  |
|  | Rate of school leavers w/o certificate | 0.01566 | 0.00568 | 0.006 | 1.578 | 0.463 | 2.710 |  |  |  |  |
| simple NB | Intercept | -4.21325 | 0.02120 | <0.001 |  |  |  | 21.00 | 1.49 | 47580.6 | <0.001 |
|  | Hospital beds/1000 population | 0.00686 | 0.00284 | 0.016 | 0.689 | 0.114 | 1.272 |  |  |  |  |
| simple NB | Intercept | -4.16931 | 0.02839 | <0.001 |  |  |  | 20.82 | 1.48 | 48652.6 | <0.001 |
|  | GPs/100,000 population | 0.00001 | 0.00043 | 0.990 | 0.001 | -0.084 | 0.086 |  |  |  |  |
| simple NB | Intercept | -4.24786 | 0.02360 | <0.001 |  |  |  | 21.55 | 1.53 | 46491.2 | <0.001 |
|  | Distance to the next pharmacy (1000m) | 0.05198 | 0.01397 | <0.001 | 5.336 | 2.518 | 8.240 |  |  |  |  |
| multiple NB | Intercept | -4.36397 | 0.05540 | <0.001 |  |  |  | 22.47 | 1.60 | 43161.4 | <0.001 |
|  | Hospital beds/1000 population | 0.01439 | 0.00445 | 0.001 | 1.449 | 0.537 | 2.378 |  |  |  |  |
|  | GPs/100,000 population | -0.00021 | 0.00079 | 0.787 | -0.021 | -0.176 | 0.134 |  |  |  |  |
|  | Distance to the next pharmacy (1000m) | 0.07611 | 0.01785 | <0.001 | 7.908 | 4.256 | 11.706 |  |  |  |  |
| Multiple NB (full model) | Intercept | -4.29707 | 0.17260 | <0.001 |  |  |  | 26,2 | 1,87 | 33619,3 | <0,001 |
|  | Unemployment rate | 0.02093 | 0.00601 | <0.001 | 2.115 | 0.919 | 3.331 |  |  |  |  |
|  | Net household income (100 Euro) | -0.01000 | 0.00708 | 0.158 | -0.995 | -2.356 | 0.402 |  |  |  |  |
|  | Rate of school leavers w/o certificate | 0.01142 | 0.00569 | 0.045 | 1.149 | 0.034 | 2.279 |  |  |  |  |
|  | Hospital beds/1000 population | 0.01082 | 0.00418 | 0.010 | 1.088 | 0.248 | 1.941 |  |  |  |  |
|  | GPs/100,000 population | -0.00093 | 0.00075 | 0.213 | -0.093 | -0.237 | 0.052 |  |  |  |  |
|  | Distance to the next pharmacy (1000m) | 0.06787 | 0.01822 | <0.001 | 7.022 | 3.242 | 10.959 |  |  |  |  |

**Table 2: Regression coefficients of the negative binomial regression model, the expected percentage change (*EPC_j_*), the dispersion parameter (*θ*), and the *χ*^2^-Test (i.e., the likelihood ratio test) for overdispersion. Outcomes: Age-standardized Sepsis incidence rates.**

| **Outcome: Age-standardized Incidence of Sepsis (explicit)** | | | | | | | | | | | |
| --- | --- | --- | --- | --- | --- | --- | --- | --- | --- | --- | --- |
| **Model** | **Predictor/Intercept** | *β* | *SE* | *p* | *EPC_j_* | 95% CI | | *θ* | *SE* | *χ*^2^ | *p* |
| simple NB | Intercept | -6.31034 | 0.03894 | < 0.001 |  |  |  | 10.18 | 0.74 | 10405.6 | <0.001 |
|  | Unemployment rate | -0.00077 | 0.00615 | 0.901 | -0.077 | -1.250 | 1.120 |  |  |  |  |
| simple NB | Intercept | -6.11847 | 0.14126 | < 0.001 |  |  |  | 10.24 | 0.75 | 10445.1 | <0.001 |
|  | Net household income (100 Euro) | -0.01088 | 0.00777 | 0.161 | -1.082 | -2.528 | 0.401 |  |  |  |  |
| simple NB | Intercept | -6.40632 | 0.04822 | < 0.001 |  |  |  | 10.29 | 0.75 | 10237.6 | <0.001 |
|  | Rate of school leavers w/o certificate | 0.01525 | 0.00762 | 0.045 | 1.537 | 0.048 | 3.060 |  |  |  |  |
| multiple NB | Intercept | -6.04303 | 0.23470 | < 0.001 |  |  |  | 10.40 | 0.76 | 10183.1 | <0.001 |
|  | Unemployment rate | -0.01656 | 0.00843 | 0.050 | -1.642 | -3.227 | -0.020 |  |  |  |  |
|  | Net household income (100 Euro) | -0.01582 | 0.01053 | 0.133 | -1.570 | -3.488 | 0.426 |  |  |  |  |
|  | Rate of school leavers w/o certificate | 0.01822 | 0.00890 | 0.041 | 1.839 | 0.075 | 3.642 |  |  |  |  |
| simple NB | Intercept | -6.35506 | 0.03095 | < 0.001 |  |  |  | 10.22 | 0.75 | 10523.3 | <0.001 |
|  | Hospital beds/1000 population | 0.00626 | 0.00417 | 0.133 | 0.628 | -0.197 | 1.470 |  |  |  |  |
| simple NB | Intercept | -6.34088 | 0.04141 | < 0.001 |  |  |  | 10.20 | 0.75 | 10555.2 | <0.001 |
|  | GPs/100,000 population | 0.00043 | 0.00063 | 0.495 | 0.043 | -0.079 | 0.167 |  |  |  |  |
| simple NB | Intercept | -6.35930 | 0.03478 | < 0.001 |  |  |  | 10.24 | 0.75 | 10701.9 | <0.001 |
|  | Distance to the next pharmacy (1000m) | 0.02952 | 0.02061 | 0.152 | 2.995 | -1.099 | 7.274 |  |  |  |  |
| multiple NB | Intercept | -6.49626 | 0.08309 | < 0.001 |  |  |  | 10.36 | 0.76 | 10328.0 | <0.001 |
|  | Hospital beds/1000 population | 0.00779 | 0.00670 | 0.245 | 0.782 | -0.550 | 2.147 |  |  |  |  |
|  | GPs/100,000 population | 0.00069 | 0.00119 | 0.565 | 0.069 | -0.164 | 0.303 |  |  |  |  |
|  | Distance to the next pharmacy (1000m) | 0.05958 | 0.02672 | 0.026 | 6.139 | 0.620 | 11.994 |  |  |  |  |
| Multiple NB (full model) | Intercept | -6.25201 | 0.27739 | < 0.001 |  |  |  | 10.48 | 0.77 | 10001.0 | <0.001 |
|  | Unemployment rate | -0.01612 | 0.00966 | 0.095 | -1.599 | -3.414 | 0.263 |  |  |  |  |
|  | Net household income (100 Euro) | -0.01108 | 0.01137 | 0.330 | -1.101 | -3.215 | 1.100 |  |  |  |  |
|  | Rate of school leavers w/o certificate | 0.01614 | 0.00916 | 0.078 | 1.627 | -0.178 | 3.475 |  |  |  |  |
|  | Hospital beds/1000 population | 0.00779 | 0.00674 | 0.248 | 0.782 | -0.562 | 2.160 |  |  |  |  |
|  | GPs/100,000 population | 0.00052 | 0.00120 | 0.668 | 0.052 | -0.183 | 0.288 |  |  |  |  |
|  | Distance to the next pharmacy (1000m) | 0.03454 | 0.02930 | 0.238 | 3.514 | -2.347 | 9.768 |  |  |  |  |
| **Outcome: Age-standardized Incidence of Sepsis (implicit)** | | | | | | | | | | | |
| **Model** | **Predictor/Intercept** | *β* | *SE* | *p* | *EPC_j_* | 95% CI | | *θ* | *SE* | *χ*^2^ | *p* |
| simple NB | Intercept | -4.27048 | 0.02327 | < 0.001 |  |  |  | 27.77 | 1.98 | 32473.2 | <0.001 |
|  | Unemployment rate | 0.01342 | 0.00368 | < 0.001 | 1.351 | 0.629 | 2.082 |  |  |  |  |
| simple NB | Intercept | -3.78380 | 0.08359 | < 0.001 |  |  |  | 28.46 | 2.03 | 33463.2 | <0.001 |
|  | Net household income (100 Euro) | -0.02267 | 0.00460 | < 0.001 | -2.242 | -3.116 | -1.354 |  |  |  |  |
| simple NB | Intercept | -4.27492 | 0.02909 | < 0.001 |  |  |  | 27.44 | 1.96 | 35531.5 | <0.001 |
|  | Rate of school leavers w/o certificate | 0.01375 | 0.00460 | 0.003 | 1.384 | 0.476 | 2.306 |  |  |  |  |
| multiple NB | Intercept | -3.88816 | 0.13949 | < 0.001 |  |  |  | 28.53 | 2.04 | 32108.4 | <0.001 |
|  | Unemployment rate | 0.00230 | 0.00502 | 0.647 | 0.230 | -0.734 | 1.208 |  |  |  |  |
|  | Net household income (100 Euro) | -0.01893 | 0.00626 | 0.003 | -1.875 | -3.057 | -0.664 |  |  |  |  |
|  | Rate of school leavers w/o certificate | 0.00392 | 0.00529 | 0.458 | 0.393 | -0.640 | 1.440 |  |  |  |  |
| simple NB | Intercept | -4.23258 | 0.01869 | < 0.001 |  |  |  | 27.11 | 1.94 | 34918.5 | <0.001 |
|  | Hospital beds/1000 population | 0.00625 | 0.00251 | 0.013 | 0.627 | 0.127 | 1.135 |  |  |  |  |
| simple NB | Intercept | -4.20522 | 0.02505 | < 0.001 |  |  |  | 26.85 | 1.91 | 36371.6 | <0.001 |
|  | GPs/100,000 population | 0.00021 | 0.00038 | 0.579 | 0.021 | -0.053 | 0.096 |  |  |  |  |
| simple NB | Intercept | -4.21865 | 0.02113 | < 0.001 |  |  |  | 26.97 | 1.92 | 36582.4 | <0.001 |
|  | Distance to the next pharmacy (1000m) | 0.01742 | 0.01251 | 0.164 | 1.757 | -0.671 | 4.251 |  |  |  |  |
| multiple NB | Intercept | -4.27295 | 0.05002 | < 0.001 |  |  |  | 27.66 | 1.98 | 34409.8 | <0.001 |
|  | Hospital beds/1000 population | 0.01218 | 0.00402 | 0.002 | 1.225 | 0.411 | 2.053 |  |  |  |  |
|  | GPs/100,000 population | -0.00067 | 0.00071 | 0.352 | -0.067 | -0.206 | 0.074 |  |  |  |  |
|  | Distance to the next pharmacy (1000m) | 0.02845 | 0.01611 | 0.077 | 2.886 | -0.260 | 6.144 |  |  |  |  |
| Multiple NB (full model) | Intercept | -4.03932 | 0.16433 | < 0.001 |  |  |  | 28,97 | 2,07 | 30719,4 | <0.001 |
|  | Unemployment rate | 0.00566 | 0.00572 | 0.323 | 0.568 | -0.548 | 1.701 |  |  |  |  |
|  | Net household income (100 Euro) | -0.01337 | 0.00674 | 0.047 | -1.328 | -2.621 | -0.003 |  |  |  |  |
|  | Rate of school leavers w/o certificate | 0.00317 | 0.00542 | 0.560 | 0.317 | -0.743 | 1.392 |  |  |  |  |
|  | Hospital beds/1000 population | 0.00984 | 0.00398 | 0.013 | 0.989 | 0.190 | 1.801 |  |  |  |  |
|  | GPs/100,000 population | -0.00093 | 0.00071 | 0.192 | -0.092 | -0.230 | 0.046 |  |  |  |  |
|  | Distance to the next pharmacy (1000m) | 0.01931 | 0.01735 | 0.266 | 1.950 | -1.448 | 5.480 |  |  |  |  |

**Table 3: Demographics of sepsis patients by explicit and implicit definition**

|  | **Explicit sepsis** | | **Implicit sepsis** | |
| --- | --- | --- | --- | --- |
| Cases, n, % | 146,985 |  | 123,6502 |  |
| Deaths, case fatality in % | 58,689 | 39.9% | 196,440 | 15.9% |
| Age in years, mean (SD), median (IQR) | 69.9 (16.1) | 74 (19) | 69.4 (21.2) | 75 (20) |
| Female gender, n, % | 60,243 | 41.0% | 580,987 | 47.0% |
| Charlson Comorbidity Index, mean (SD), median (IQR) | 2.2 (1.5) | 2 (2) | 2.1 (0.5) | 2 (2) |
| Comorbidities |  |  |  |  |
| Diabetes, n, % | 48,811 | 33.2% | 372,334 | 30.1% |
| Chronic pulmonary disease, n, % | 24,194 | 16.5% | 316,266 | 25.6% |
| Renal disease, n, % | 49,191 | 33.5% | 434,938 | 35.2% |
| Congestive heart failure and myocardial infarction, n, % | 60,427 | 41.1% | 477,577 | 38.6% |
| Cancer, n, % | 27,790 | 18.9% | 186,211 | 15.1% |
| Dementia or cerebrovascular disease, n, % | 31,053 | 21.1% | 280,385 | 22.7% |
| Liver disease, n, % | 13,467 | 9.2% | 73,442 | 5.9% |
| HIV or AIDS, n, % | 297 | 0.2% | 1,407 | 0.1% |
| Proportion of septic shock, n, % | 44,657 | 30.4% | 44,657 | 3.6% |
| Surgical treatment, n, % | 63,122 | 42.9% | 326,681 | 26.4% |
| ICU admission, n, % | 79,194 | 54.2% | 309,153 | 25.1 % |
| Hospital length of stay [days], mean (SD), median (IQR) | 22.3 (25.0) | 15 (22) | 15.8 (1.9) | 11 (14) |
| Discharge to hospice, n, % | 247 | 0.2% | 3,016 | 0.2% |

**Table 4A:** Mean age, socioeconomic status and health care capacity among German federal states

| Predictor | M | Median | SD | Min | Max |
| --- | --- | --- | --- | --- | --- |

| Mean Age | 44.50 | 44.11 | 1.74 | 41.73 | 47.21 |
| --- | --- | --- | --- | --- | --- |
| Unemployment rate | 7.11 | 7.15 | 2.10 | 3.50 | 10.50 |
| Net household income (100 Euro) | 17.54 | 17.35 | 1.63 | 15.26 | 20.22 |
| Rate of school leavers w/o certificate | 6.77 | 6.44 | 1.54 | 5.06 | 9.67 |
| Hospital beds/1000 population | 6.29 | 6.28 | 0.73 | 5.11 | 7.64 |
| GPs/100,000 population | 64.32 | 62.38 | 8.87 | 55.04 | 85.45 |
| Distance to the next pharmacy (1000m) | 1.31 | 1.43 | 0.57 | 0.40 | 2.38 |

**Table 4B:** Mean age, socioeconomic status and health care capacity by German federal states

| Federal state | Mean Age | Unemployment rate (%) | Nethaushold income (100 Euro) | Rate of school leavers w/o certificate | Hospital beds/1000 population | GPs/100,000 population | Distance to next pharmacy (1000m) |
| --- | --- | --- | --- | --- | --- | --- | --- |
| Schleswig-Holstein | 44.63 | 6.3 | 18.44 | 6.62 | 5.57 | 57.36 | 1.58 |
| Hamburg | 41.73 | 7.1 | 20.22 | 5.85 | 6.93 | 79.37 | 0.51 |
| Lower Saxony | 44.01 | 6.0 | 17.52 | 5.10 | 5.28 | 56.80 | 1.53 |
| Bremen | 43.24 | 10.5 | 17.18 | 6.25 | 7.64 | 85.45 | 0.49 |
| North Rhine Westphalia | 43.59 | 7.7 | 18.00 | 5.32 | 6.69 | 62.50 | 0.88 |
| Hessen | 43.34 | 5.3 | 18.66 | 5.40 | 5.82 | 57.12 | 1.09 |
| Rhineland Palatinate | 44.21 | 5.1 | 18.50 | 5.92 | 6.21 | 57.92 | 1.51 |
| Baden Wurttemberg | 42.94 | 3.8 | 19.89 | 5.15 | 5.11 | 59.19 | 1.11 |
| Bavaria | 43.26 | 3.5 | 19.95 | 5.06 | 5.89 | 59.09 | 1.50 |
| Saarland | 45.72 | 7.2 | 17.10 | 6.98 | 6.51 | 68.13 | 1.01 |
| Berlin | 42.22 | 9.8 | 16.31 | 8.36 | 5.63 | 75.92 | 0.40 |
| Brandenburg | 46.55 | 8.0 | 16.16 | 7.29 | 6.13 | 55.04 | 2.12 |
| Mecklenburg-West Pomerania | 46.41 | 9.7 | 15.26 | 9.44 | 6.39 | 63.20 | 2.38 |
| Saxony | 46.31 | 7.5 | 16.00 | 8.40 | 6.35 | 67.10 | 1.36 |
| Saxony-Anhalt | 47.21 | 9.6 | 15.57 | 9.67 | 7.11 | 62.25 | 1.76 |
| Thuringia | 46.65 | 6.7 | 15.84 | 7.53 | 7.35 | 62.65 | 1.76 |

**Table 5: Likelihood tests of the reduced NB regression models either without indicators of regional socioeconomic deprivation or without health care indicators against the full NB regression with all predictors.**

| **Sepsis case definition** | **Omitted set of indicator variables** | **Log. Likelihood reduced model** | **Log. Likelihood full model** | **df** | **χ^2^** | **p value** | **Δ Pseudo-R^2^** |
| --- | --- | --- | --- | --- | --- | --- | --- |
| implicit | Socioeconomic deprivation | -3057.5 | -3026.9 | 3 | 61.11 | < 0.001 | 0.142 |
| implicit | Health care capacity | -3039.6 | -3026.9 | 3 | 25.44 | < 0.001 | 0.062 |
| explicit | Socioeconomic deprivation | -2385.6 | -2378.8 | 3 | 13.45 | 0.004 | 0.033 |
| explicit | Health care capacity | -2383. 7 | -2378.8 | 3 | 9.64 | 0.022 | 0.024 |
| age-standardized implicit | Socioeconomic deprivation | -3008.9 | -2999.8 | 3 | 18.16 | < 0.001 | 0.044 |
| age-standardized implicit | Health care capacity | -3003.8 | -2999.8 | 3 | 7.91 | 0.048 | 0.020 |
| age-standardized explicit | Socioeconomic deprivation | -2356.7 | -2354.3 | 3 | 4.79 | 0.188 | 0.012 |
| age-standardized explicit | Health care capacity | -2356.5 | -2354.3 | 3 | 4.48 | 0.214 | 0.011 |

**References**

1. Pebesma EJ, Bivand RS (2005) Classes and methods for spatial data in R. R News 5 (2). In:<https://cran.r-project.org/doc/Rnews/>.

2. Bivand RS, Pebesma EJ, Gomez-Rubio V (2013) Applied spatial data analysis with R. Springer, New Yok (NJ), US
