# Supplementary material for: Association between sepsis incidence and regional socioeconomic deprivation and health care capacity in Germany – an ecological study
Source: BMC Public Health. 2021 Sep 7;21:1636. doi: 10.1186/s12889-021-11629-4 (PMC8424852; doi:10.1186/s12889-021-11629-4)
Supplement: Supplementary file 3 — Additional file 3. Supplement to Methods/Statistical analyses. Supplementary file 3 includes a supplementary statistical analyses section. [file 12889_2021_11629_MOESM3_ESM.pdf]

Supplementary File 3

Association between sepsis incidence and regional socioeconomic deprivation and health care capacity in Germany – An ecological study

Dr. Norman Rose[1,2], Dr. Claudia Matthäus-Krämer[1], Dr. Daniel Schwarzkopf[2,3], Prof. André Scherag[4], Dr. Sebastian Born[1,2], Prof. Konrad Reinhart[5], Dr. Carolin Fleischmann-Struzek[1,2]

1 Center for Sepsis Control and Care, Jena University Hospital, Bachstraße 18, 07743 Jena, Germany

2 Institute of Infectious Diseases and Infection Control, Jena University Hospital, Am Klinikum 1, 07747 Jena, Germany

3 Department for Anesthesiology and Intensive Care Medicine, Jena University Hospital,

Am Klinikum 1, 07740 Jena, Germany,

4 Institute of Medical Statistics, Computer and Data Sciences, Jena University Hospital, Bachstraße 18, 07743 Jena, Germany

5 Department of Anesthesiology and Intensive Care Medicine, Charité Universitätsmedizin Berlin, Charitéplatz 1, 10117 Berlin, Germany

Statistical Analyses

The simple and multiple negative binomial (NB) regression model was used, with the number of sepsis cases per district as the outcome variable (denoted by the variable Y). The NB model was preferred to the standard Poisson Model due to the problem of overdispersion in our data. The logarithm of the number of inhabitants per district was included as an offset variable in the model to adjust for varying population sizes across districts. The NB model with $k \geq 1$ predictor variables X_j (with $j = 1, \ldots, k$) can be written as

$$E(Y \mid \boldsymbol{X}) = \exp\left(\beta_0 + \sum_{j=1}^{k} \beta_j X_j + \log(N) + \varepsilon\right) \tag{1}$$

The conditional variance function is

$$Var(Y \mid \boldsymbol{X}) = E(Y \mid \boldsymbol{X})\left[1 + \theta^{-1} E(Y \mid \boldsymbol{X})\right] \tag{2}$$

This parameterization of the model is also referred to as the NB1 model in the literature[1]. θ is the dispersion parameter, which is defined as the precision (e.g. the inverse variance) of the exponential function of the random effects term ε. Hence $\theta^{-1} = Var[\exp(\varepsilon)]$. Note that $Var[\exp(\varepsilon)]$ approach zero if θ approaches infinity. In this case the Poisson regression model results with the equality $E(Y \mid \boldsymbol{X}) = Var(Y \mid \boldsymbol{X})$. We tested for overdispersion by a statistical comparison of the NB model and the more restricted Poisson model using the likelihood ratio test, with the Null hypothesis H_0: $Var[\exp(\varepsilon)] = 0$ (e.g. no overdispersion). We used the R-function glm.nb from the MASS package[2] for fitting the NB model, and the glm of the base distribution of R[3] function for estimating the Poisson regression model.

Pseudo-R^2 for the Negative Binomial Regression Models

We provide Nagelkerkes pseudo-R^2 as a standardized measure of the strength of the stochastic relationship between the outcome variable Y (i.e., the incidence) and one or more predictor variables. In our case the computation of the pseudo-R^2 was computed based on two likelihood functions. First, likelihood of the NB model with the predictor variables $X_1, \ldots, X_k$ and the offset variable $\log(N)$, and second, the NB model that only includes the intercept and the offset variable $\log(N)$.

Estimated Expected Change

The regression coefficients β_j in the NB regression model are differences in the logarithm of the expected numbers of events if the predictor X_j increases by one unit. As differences at the logarithmic scale are hard to interpret, we converted the regression coefficients to an expected change in the number of cases if the predictor X_j increases by one unit (EC_j). Positive numbers of EC_j represent an increase in sepsis cases while negative values means that the incidence decreases if X_j increases. The EC_j can be derived from Equation 1 with a single predictor variable X_j:

$$\begin{aligned}
E(Y \mid X_j = x_j + 1) - E(Y \mid X_j = x_j) &= \exp\left(\beta_0 + \beta_j\left(x_j + 1\right) + \ln(N) + \varepsilon\right) - \exp\left(\beta_0 + \beta_j x_j + \ln(N) + \varepsilon\right) \\
&= \exp\left(\beta_0 + \beta_j x_j + \beta_j + \ln(N) + \varepsilon\right) - \exp\left(\beta_0 + \beta_j x_j + \ln(N) + \varepsilon\right) \\
&= \exp\left(\beta_0 + \beta_j x_j + \ln(N) + \varepsilon\right)\exp\left(\beta_1\right) - \exp\left(\beta_0 + \beta_j x_j + \ln(N) + \varepsilon\right) \\
&= \left(\exp\left(\beta_j\right) - 1\right)\exp\left(\beta_0 + \beta_j x_j + \ln(N) + \varepsilon\right) \\
&= \left(\exp\left(\beta_j\right) - 1\right) E(Y \mid X_j = x_j)
\end{aligned} \tag{3}$$

In order to get the EC_j per 100.000 population units, Equation 3 must be multiplied by 100.000. Note that the term $\exp(\beta_j) - 1$ is the expected proportional change (EPC_j):

$$\exp(\beta_j) - 1 = \frac{E(Y \mid X_j = x_j + 1) - E(Y \mid X_j = x_j)}{E(Y \mid X_j = x_j)} \tag{4}$$

Hence, 100 · EPC_j is the expected percentage change in the number of cases if X_j increases by one unit. While the EPC_j is constant for all values x_j, EC_j is a function with different numbers depending on the value x_j. In order to provide meaningful estimates, we computed EC_j if the mean $\bar{x}_j$ of the predictor X_j increases by one unit:

$$\left[E(Y \mid X_j = \bar{x}_j + 1) - E(Y \mid X_j = \bar{x}_j)\right] = \left(\exp(\beta_j) - 1\right) E(Y \mid X_j = \bar{x}_j) \tag{5}$$

Note that the Equations 3 to 5 were derived for the case of the NB regression with one predictor variable X_j. However, the EC_j as well as the EPC_j can also be computed based on partial regression coefficients of a multiple NB regression with $k \geq 2$ predictor variables. In this case the EC_j and the EPC_j are computed under statistical control of the remaining predictor variables X_i, with $i \neq j$, in the model. Therefore we denote these quantities as the adjusted EC_j and the adjusted EPC_j. The latter is defined as

$$\exp(\beta_j) - 1 = \frac{E(Y \mid \boldsymbol{Z} = z, X_j = x_j + 1) - E(Y \mid \boldsymbol{Z} = z, X_j = x_j)}{E(Y \mid \boldsymbol{Z} = z, X_j = x_j)}, \tag{6}$$

with $\boldsymbol{Z}$ the vector of all covariates X_i, with $i \neq j$. The adjusted EPC_j is a constant but EC_j is a function depending not only on X_j but also from the values of the other predictor variables $\boldsymbol{Z}$. Analogous to the EC_j we computed the adjusted EC_j given that the mean $\bar{x}_j$ of the predictor X_j increases by one unit and the other predictors are constant at their means $\boldsymbol{Z} = \bar{\mathbf{z}}$. Hence the adjusted EC_j is defined as

$$\left[E(Y \mid \boldsymbol{Z} = \bar{z}, X_j = \bar{x}_j + 1) - E(Y \mid \boldsymbol{Z} = \bar{z}, X_j = \bar{x}_j)\right] = \left(\exp(\beta_j) - 1\right) E(Y \mid \boldsymbol{Z} = \bar{z}, X_j = \bar{x}_j) \tag{7}$$

95% confidence intervals of the estimated EPC_j, adjusted EPC_j, EC_j and the adjusted EC_j are obtained by inserting the confidence limits of β_j into Equations 4 to 7.

References

1. Greene W. Functional forms for the negative binomial model for count data. *Economics Letters.* 2008;99(3):585-590.
2. Venables WN, Ripley BD. *Modern Applied Statistics with S.* 4 ed. New York, NJ: Springer; 2002.
3. *R Core Team. R: A language and environment for statistical computing* [computer program]. Vienna, Austria: R Foundation for Statistical Computing; 2019.
